# Local and systemic cytokine signatures for outcome prediction after transarterial chemoembolization in hepatocellular carcinoma

**DOI:** 10.7150/thno.130031

**Published:** 2026-04-16

**Authors:** Emine Y. Yilmaz, Robin Schmidt, Luisa Heidemann, Yubei He, Yu Liu, Ornela Sulejmani, Dominik N. Müller, Andreas Wilhelm, Timo A. Auer, Charlie A. Hamm, Salma A. S. Abosabie, Sara A. Abosabie, Bernhard Gebauer, Lynn J. Savic

**Affiliations:** 1 Charité - Universitätsmedizin Berlin, Campus Virchow Klinikum, Department of Radiology, Germany.; 2 Experimental Clinical Research Center (ECRC) at Charité - Universitätsmedizin Berlin and Max-Delbrück-Centrum für molekulare Medizin (MDC), Germany.; 3 Universitätsmedizin Göttingen, Institute for Clinical and Interventional Radiology, Göttingen, Germany.; 4 Max Delbrück Center for Molecular Medicine in the Helmholtz Association, Berlin, Germany.; 5 Charité - Universitätsmedizin Berlin, Corporate Member of Freie Universität Berlin and Humboldt-Universität zu Berlin, Berlin, Germany.; 6 DZHK (German Centre for Cardiovascular Research), partner site Berlin, Germany.; 7 CheckImmune GmbH, 13353 Berlin, Germany.; 8 Berlin Institute of Health at Charité- Universitätsmedizin Berlin, Germany.; 9 Department of Radiology and Biomedical Imaging, Yale University School of Medicine, New Haven, CT, USA.; 10 Institute of Experimental Biomedicine, University Hospital Wuerzburg, Würzburg, Germany.; 11 Rudolf Virchow Center for Integrative and Translational Bioimaging, Julius Maximilians University of Wuerzburg, Würzburg, Germany.

**Keywords:** hepatocellular carcinoma, chemoembolization, cytokines, tumor interstitial fluid, immune biomarkers, lipiodol deposition

## Abstract

Purpose: Conventional transarterial chemoembolization (cTACE) is a guideline-approved therapy for unresectable hepatocellular carcinoma (HCC). This study aimed to characterize systemic and local cytokine changes induced by cTACE, and to evaluate their correlation with treatment response, Lipiodol deposition, and overall survival (OS).

Experimental Design: In this prospective single-center study, 46 patients with unresectable HCC underwent either cTACE followed by interstitial brachytherapy (iBT) after 24h (n = 23) or iBT alone (n = 23). Peripheral blood samples were obtained before treatment in both groups and 24h post-cTACE in the cTACE/iBT group. Additionally, tumor interstitial fluid (TIF) was isolated from tumor biopsies that were collected prior to iBT from untreated tumors (iBT group) or 24h post-cTACE (cTACE/iBT group). Serum and TIF cytokines were quantified using multiplex assays and correlated with radiologic response at 8-week MRI, Lipiodol patterns on CT 24h post-cTACE, and OS.

Results: Post-cTACE, serum IFN-γ, MCP-1, TNF-α, MIP-1α, IL-17, IL-8, IL-4, and IL-5 significantly decreased, while IL-6 increased (p < 0.005). TIF analysis showed higher IL-17 in untreated tumors compared to post-cTACE (p = 0.038), but differences were modest. In the iBT group, responders had elevated TIF IL-8 (p = 0.0103) and VEGF-A (p = 0.0185) prior to treatment, whereas in the cTACE/iBT group, responders exhibited lower TIF bFGF post-cTACE than non-responders (p = 0.0449). Elevated baseline serum IL-6 (p = 0.052), IL-8 (p = 0.036), and IFN-γ (p = 0.0508) were associated with shorter OS, while higher TIF IFN-γ (p = 0.051) correlated with improved OS. Baseline serum IL-1β (p = 0.0266) and post-cTACE serum level of VEGF-A (p = 0.0318) were higher in patients with homogeneous Lipiodol deposition in tumors, which correlated with longer OS.

Conclusions: cTACE induces distinct systemic and local immune alterations that influence patient outcomes. Specifically, elevated serum IL-6 predicted poor survival, while homogeneous Lipiodol deposition marks favorable immune modulation and outcomes. Combining cytokine profiling with imaging biomarkers may enable improved, personalized treatment strategies in HCC.

## Introduction

Hepatocellular carcinoma (HCC) is the third most common cause of cancer-related deaths worldwide, presenting a major global health burden 1. Locoregional therapies (LRTs), including conventional transarterial chemoembolization (cTACE), are minimally invasive, guideline-approved treatment strategies for unresectable HCC, achieving local tumor control with relatively low complication rates 2,3. However, they frequently fail to achieve long-term tumor eradication, particularly in larger or multifocal tumors, leading to high local recurrence rates 4.

Despite the widespread use of cTACE and given the absence of widely applicable and clinically reliable prognostic scores, an unmet need remains for biologically driven biomarkers that can predict treatment response, guide patient selection, and prevent ineffective LRT in favor of timely systemic approaches 5,6.

Beyond its direct ischemic and cytotoxic effects, cTACE also plays a critical immunomodulatory role by altering the composition of the tumor microenvironment (TME) and influencing both pro-tumorigenic and anti-tumorigenic immune pathways 7. This immune-modulating capacity has gained increasing attention, particularly in the era of combination therapies, where systemic immuno-therapy and anti-angiogenic agents are combined with LRTs. In recent clinical trials, such as EMERALD-1, LEAP-012, and EMERALD-3, the combination of TACE with immune checkpoint inhibitors (ICIs) and vascular endothelial growth factor (VEGF) inhibitors demonstrated improved progression-free survival of patients with unresectable HCC 8-11.

However, a major challenge in optimizing combination therapies lies in patient selection, therapeutic sequencing and timing. Currently, treatment decisions are not biologically driven because reliable biomarkers are lacking that characterize the TME as favorably immunopermissive or immunoevasive. Consequently, tumors are largely treated uniformly, resulting in ineffective treatments, tumor recurrence, and the development of therapy resistance in subsets of patients.

Meanwhile, the inherent complexity of the TME as well as immune responses to treatment makes therapeutic responses difficult to anticipate 12,13. Cytokines such as interleukin-6 (IL-6), interleukin-8 (IL-8), and interferon-gamma (IFN-γ) are central regulators of tumor progression in HCC 14. IL-6 promotes tumor growth and therapy resistance through pro-inflammatory and pro-angiogenic signaling 15-17, while IL-8 supports angiogenesis, immune suppression, and tumor relapse 18-22. IFN-γ, produced by CD8+ and CD4+ T cells and NK cells, has paradoxical roles in cancer. While acute IFN-γ signaling is essential for anti-tumor immunity and effective immunotherapy responses, prolonged or dysregulated signaling can drive immune evasion and therapy resistance 23-28.

Recent retrospective or small prospective analyses of selected circulating cytokines suggested an association between systemic inflammatory markers and outcomes 14-16. However, the extent to which circulating biomarkers reflect treatment-induced changes within the TME remains unclear. Within the TME, the tumor interstitial fluid (TIF) is increasingly recognized as a critical component hosting cytokines and other humoral factors that could influence the response to treatment 29-31. Additionally, the composition and dynamics of TIF have been shown to affect drug delivery, therapy resistance, and metastatic potential in multiple cancer entities, including HCC 32,33.

This prospective study was designed to test the hypothesis that cTACE induces distinct and measurable humoral immune alterations, and that these effects differ between the systemic circulation and the local TME. Therefore, we comprehensively profiled cytokine signatures in paired peripheral blood (serum) and TIF samples from patients with unresectable HCC undergoing cTACE. Longitudinal serum sampling in cTACE-treated patients enabled the characterization of dynamic systemic immune modulation, while tumor biopsies from untreated HCC and HCC post-cTACE allowed discrimination of treatment-induced immune alterations from baseline immune signatures. We further explored whether cytokine patterns can help predict patient outcomes by correlating them with imaging-based tumor response and overall survival (OS).

## Methods

### Study design and patient cohort

This prospective single-center study was approved by the local ethics committee and registered in the German Clinical Trials Register (DRKS00026994) and conducted in accordance with the Declaration of Helsinki. Written informed consent was obtained from all participants. Between February 2022 and August 2023, 46 consecutive patients with unresectable HCC at an early or intermediate stage and without prior locoregional therapy were enrolled, who underwent either cTACE followed by CT-guided high-dose rate brachytherapy (iBT) after 24 hours (cTACE/iBT group) or iBT alone (iBT group). Peripheral blood and tumor biopsies were collected for cytokine and angiogenic factor analysis. In the cTACE/iBT group, blood samples were obtained prior to and 24 hours after cTACE, and tumor biopsies were collected 24 hours after cTACE and before iBT. In the iBT group, both blood samples and tumor biopsies were obtained prior to iBT from untreated patients and thus, served as untreated controls. Biopsies from the cTACE/iBT group are referred to as “post-cTACE,” while those from the iBT-only group are referred to as “untreated.” Treatment allocation was not randomized but determined by the multidisciplinary tumor board based on lesion size, location, and vascularization.

Diagnosis of HCC was established by imaging using the Liver Imaging Reporting and Data System (LI-RADS) or by histopathology. Eligibility criteria included: measurable, non-infiltrative HCC; age ≥18 years; ineligibility for surgical resection or liver transplantation; Eastern Cooperative Oncology Group (ECOG) performance status 0-2; Child-Pugh class A or B; and Barcelona Clinic Liver Cancer (BCLC) stage A or B. Prior systemic therapy, including immunotherapy, was allowed if completed ≥4 weeks before enrollment; two patients in this cohort had received such therapy.

**Figure 1:**
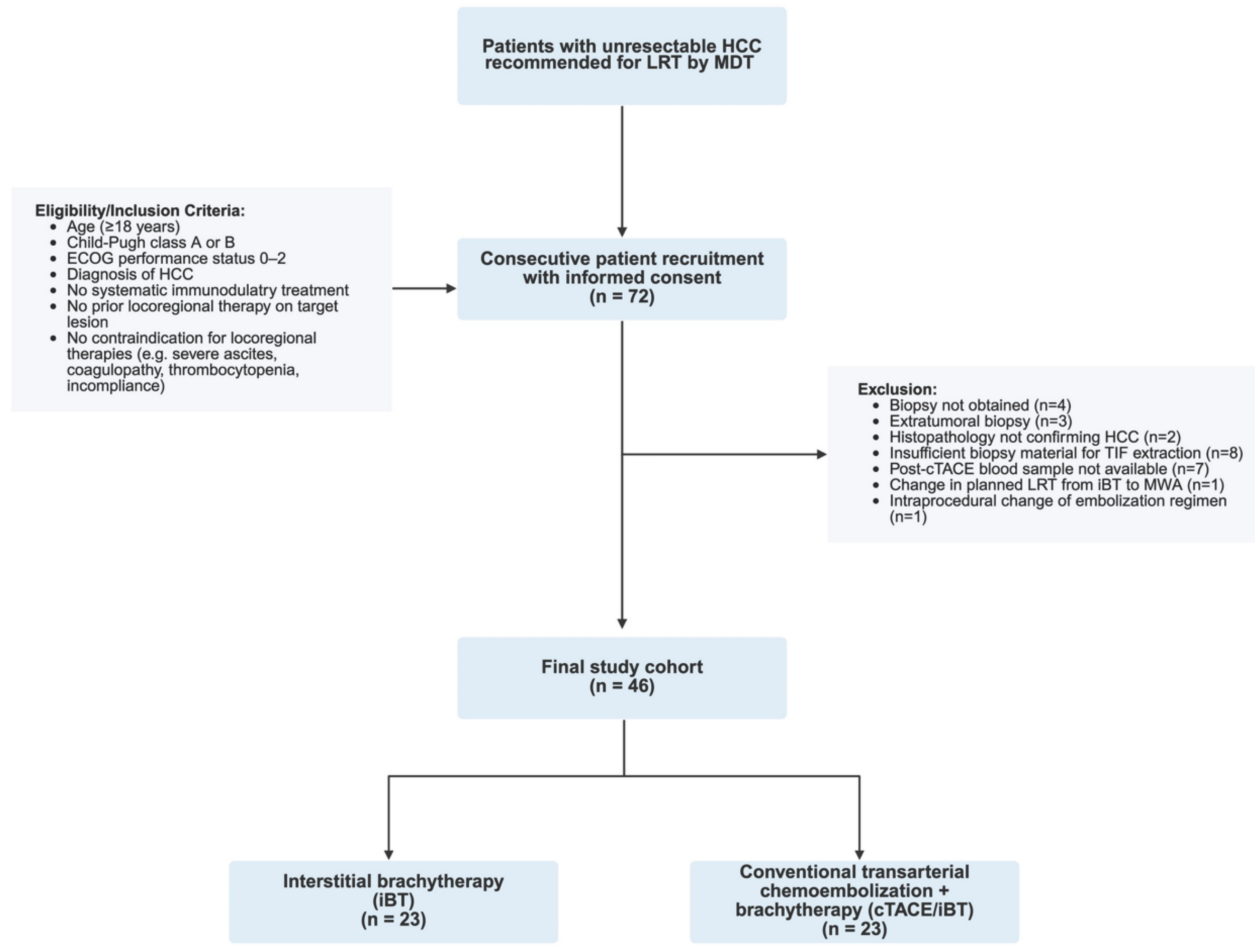
** Study flow chart.** Legend: The diagram illustrates patient screening, inclusion, exclusion, and allocation to treatment groups in this prospective single-center study. Hepatocellular carcinoma (HCC), locoregional therapy (LRT), multidisciplinary tumor board (MDT), Eastern Cooperative Oncology Group (ECOG).

**Figure 2:**
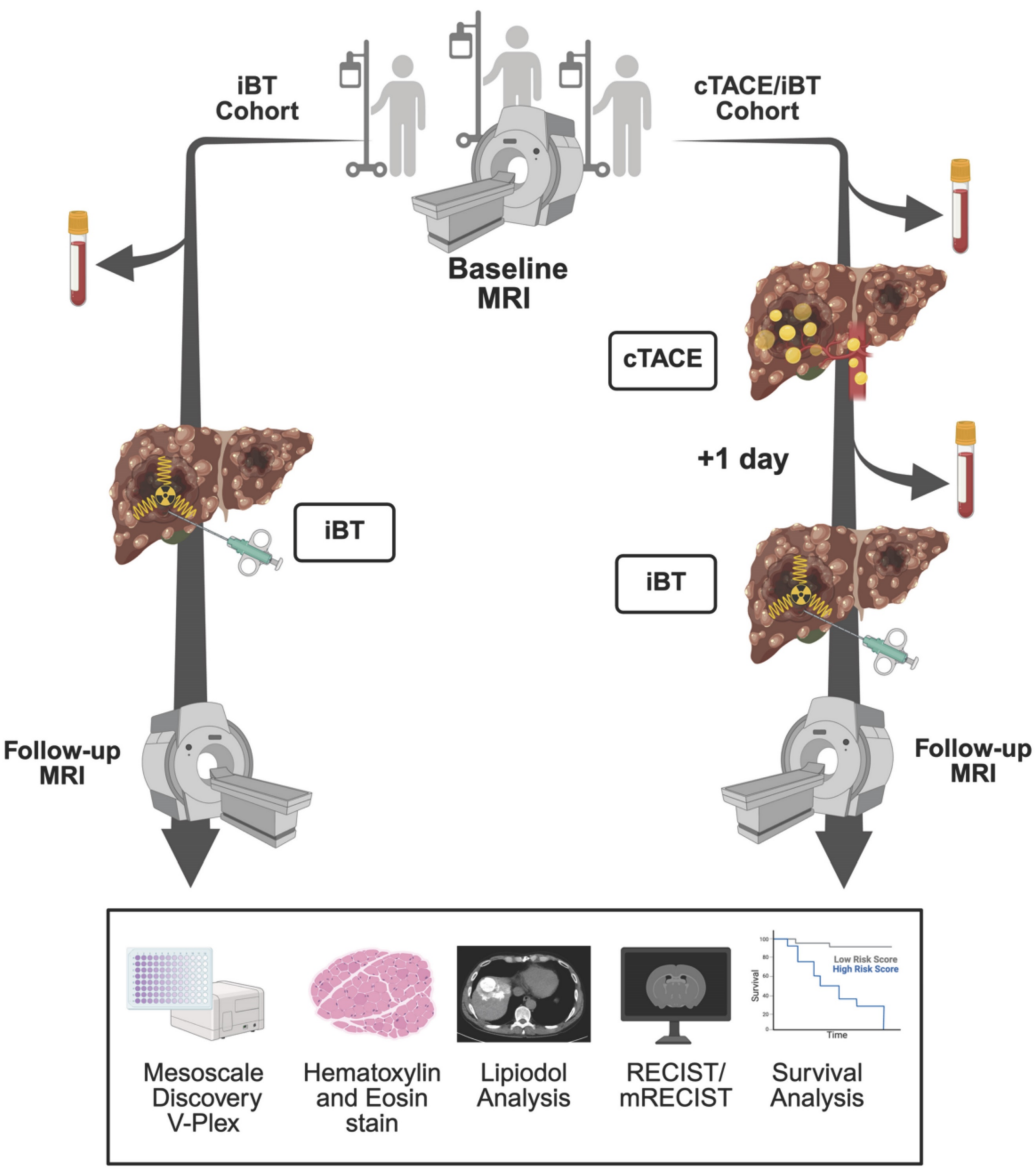
** Study Design.** Legend: Schematic overview of patient allocation and workflow. In the interstitial brachytherapy (iBT) cohort (left), patients underwent baseline MRI, followed by blood and tumor biopsy collection before iBT and subsequent follow-up imaging at eight weeks. In the conventional transarterial chemoembolization (cTACE)/iBT group (right), baseline MRI (or CT in two patients) was followed by cTACE with Lipiodol delivery; blood and tumor biopsies were collected 24 hours later immediately prior to iBT, with follow-up imaging at 8 weeks. Blood samples and biopsies were analyzed by multiplex cytokine assays (Mesoscale Discovery V-Plex). Additionally, tumor biopsies were analyzed by H&E staining, and Lipiodol deposition was assessed on non-enhanced CT one day after cTACE. Tumor response was assessed, and overall survival was recorded.

All participants underwent laboratory testing to assess liver function (serum albumin, prothrombin time, total bilirubin, aspartate aminotransferase, alanine aminotransferase) and hematologic parameters (hemoglobin, leukocyte count, platelet count) 24 hours before and after the investigational LRT. Dynamic contrast-enhanced magnetic resonance imaging (MRI) or computed tomography (CT) was acquired within 30 days prior to LRT and at 8-12 weeks post-treatment for imaging feature extraction and tumor response assessment. Patient screening, eligibility assessment, exclusion reasons, and final cohort allocation are summarized in the flowchart shown in Figure 1. The overall study workflow, including patient allocation, sample collection, and imaging timeline is illustrated in Figure 2.

### Study endpoints

The primary endpoint was the effect of cTACE on serum cytokine levels, assessed longitudinally within the cTACE/iBT group. The secondary endpoint was the effect of cTACE on TIF cytokine levels, evaluated by comparing post-cTACE biopsies with untreated biopsies. Tertiary endpoints included: (i) associations of serum and TIF cytokines with tumor response stratified by treatment group; (ii) associations of serum and TIF cytokines with Lipiodol deposition patterns after cTACE (cTACE/iBT group only); (iii) OS comparison between cTACE/iBT and iBT groups; (iv) OS stratified by baseline serum and TIF cytokine levels; and (v) multivariate analysis to identify predictive biomarkers for OS, including serum and TIF cytokines, stratified by treatment group.

### Locoregional therapies

cTACE was performed under fluoroscopic guidance according to standard institutional protocols, delivering an emulsion of doxorubicin and mitomycin C with ethiodized oil (Lipiodol, Guerbet, France) 1:2 to the tumor-feeding artery until near stasis, as described earlier. iBT was performed using CT-guided catheter placement and high-dose-rate afterloading brachytherapy with Iridium-192 (GammaMedplus iX, Varian Medical Systems, Palo Alto, CA, USA; RRID: N/A), following published guidelines 34-36. The procedural details, including equipment specifications, drug preparation, dosimetry, and embolization criteria are provided in the *Supplementary Methods.*

### Sample collection and processing

Blood collection and serum isolation: Peripheral blood samples were collected in serum separator tubes (SST; yellow-top Vacutainer®, BD Biosciences, Franklin Lakes, NJ, USA) and centrifuged at 1000 rpm for 10 minutes to obtain serum, which was subsequently aliquoted and stored at -80 °C until further analysis.

**Biopsy collection and interstitial fluid isolation:** Tumor biopsies were obtained from the target lesion for treatment. In case of multiple target lesions, biopsies were collected from the largest target lesion. The previously published elution method was adapted and used to isolate the interstitial fluid from freshly obtained tumor samples 32,33. Briefly, after weighing, samples were inserted in an Eppendorf tube (Eppendorf AG, Hamburg, Germany). A total of 0.4 - 0.8 mL HEPES buffer (1M) (N-2-hydroxye-thylpiperazine-N-2-ethane sulfonic acid; Sigma-Aldrich, St. Louis, MO, USA) was added to the biopsy, and elution of cytokines from the biopsy into the buffer took place at room temperature for 3 hours on a 50-rpm shaker. After centrifugation at 1.5 rpm for 5 minutes, the extracted fluid was transferred to fresh Eppendorf tubes with a syringe and filtered through Syringe filters (ROTILABO® MCE, 0.45 m; Carl Roth GmbH, Karlsruhe, Germany) using a Paster pipe. The collected TIF was kept at -80°C for further analysis. After the elution procedure, the remaining tumor sample was placed in 4% formalin for further histological analysis to confirm tumor origin.

**Immunoassay:** A total of 15 cytokines, chemokines, and angiogenic factors were analyzed in both TIF and blood samples using customized Meso scale (MSD) V-PLEX multiplex kits (Meso Scale Discovery, Rockville, MD, USA; Research Resource). The panel included proinflammatory (IFN-γ, IL-1β, IL-2, IL-6, IL-8, IL-17A, monocyte chemoattractant protein-1 (MCP-1), macrophage inflammatory protein-1 Alpha (MIP-1α), tumor necrosis factor-alpha (TNF-α), anti-inflammatory (IL-4, IL-5, IL-10, IL-13), and pro-angiogenic factors (vascular endothelial growth factor-A (VEGF-A), basic fibroblast growth factor (bFGF)). Data were analyzed using the MSD Discovery Workbench 4.0 software. Cytokine concentrations in TIF were normalized to biopsy mass using weight-based correction as described previously 29 and calculated using the following formula to account for the assumption that 50% of the tissue weight is water: Expected pg/mL in total tissue water = (cytokine concentration in elution volume * elution volume) / (biopsy sample weight in grams * 0.5).

### Imaging and image analysis

Target tumor response was assessed after each completed treatment on the 8-week follow-up imaging dataset according to the Response Evaluation Criteria In Solid Tumors version 1.1 (RECIST 1.1), the modified (m)RECIST criteria, and the Liver Imaging Reporting and Data System (LI-RADS).

In the cTACE/iBT group, Lipiodol distribution was assessed on non-contrast-enhanced CT acquired 24 h post-cTACE. Assessment focused on (a) the homogeneity of intratumoral Lipiodol deposition and (b) the distribution between target and non-target regions. Lipiodol deposition (a) was classified as homogeneous if ≥ 85% of the tumor volume demonstrated medium-to-high attenuation, and as heterogeneous if < 85% of the tumor was covered or if only low attenuation was observed. Lipiodol distribution (b) was further categorized as tumor-only (confined to the tumor) or segmental (extending into adjacent liver segments). Segmental deposition was further subdivided into single segment or multiple segments, depending on the extent of spread 37-39. Image analysis was performed by two readers with 3 and 10 years of experience in abdominal imaging based on consensus. Detailed MR and CT imaging protocols, signal intensity measurements, and region of interest (ROI) analysis are described in the Supplementary Methods.

### Statistics

Sample size calculation was performed prior to study initiation. Serum IL-6 was selected for sample size calculation since it is the most consistently reported cytokine in previous studies of locoregional therapy. Published data show early IL-6 increases of ~20 pg/mL after TACE, with SD_diff ≈ 34 pg/mL at 24 h, yielding a required sample size of 23 patients (paired t-test, α=0.05, power=80%) 14. An additional 23 iBT-only patients were included as untreated controls for analyses of TIF (secondary endpoint), assuming local cytokine changes would be at least comparable to, and possibly greater than those observed in serum.

Baseline characteristics were analyzed using descriptive statistics including mean, standard deviation or median and range for continuous variables. For categorical variables, absolute and relative frequencies were reported. Between-group comparisons of continuous variables were performed using the Mann-Whitney U test. Categorical variables were compared using Fisher's exact test due to small sample sizes in individual categories. Baseline associations between clinical parameters, liver function metrics, tumor characteristics, and baseline serum cytokine levels were explored using Pearson correlation analysis. The cytokine analysis and statistics were explorative using the Wilcoxon matched-pairs signed rank test or Mann-Whitney test. Survival analyses included Kaplan-Meier estimates with the log-rank test to assess differences between groups. Survival time was calculated in months from the date of LRT to death or last follow-up. Cytokine cut-off values for Kaplan-Meier analyses were defined using the median concentration within the respective study cohort. Six- and twelve-month survival estimates were additionally reported.

Univariate and multivariate Cox proportional hazards regression analyses were performed incorporating clinical, laboratory, imaging, and immunoassay parameters to identify prognostic factors. Results were expressed as hazard ratios (HR) with corresponding 95% confidence intervals (CI) and p-values. Given the exploratory biomarker setting, variables showing an association with OS at p < 0.1 in univariate Cox regression analyses were considered for inclusion in multivariate models. Multivariate model construction additionally accounted for clinical relevance, potential collinearity, and the limited number of events to avoid overfitting. In multivariate analyses, statistical significance was defined as p < 0.05. Statistical analyses and modeling were conducted using Python 3.8, GraphPad Prism (Version 9.5, GraphPad, San Diego, CA), and R software.

## Results

### Patient baseline characteristics

The cohort consisted of 36 men and ten women with a mean age of 71.4 ± 9.5 years. There were 43 patients classified as Child-Pugh A and 3 as B, with 31 in Barcelona Clinic Liver Cancer (BCLC) stage A and 15 in stage B. Overall, 29 had a single tumor, 13 had two tumors, and four had three tumors, with a mean tumor size of 41.1 ± 23.1 mm and a mean target tumor size of 33.7 ± 22.6 mm. Based on LI-RADS, 8 tumors were classified as LR-4, and 36 as LR-5; LI-RADS classification was not possible in 2 patients due to imaging limitations but diagnosis was confirmed by histopathological examination. When stratified by treatment group, patients in the cTACE/iBT group more frequently presented with BCLC B (57% vs. 9%, p = 0.0012) and had a higher proportion of LR-5 lesions (p = 0.0459) than the iBT group. In addition, the total diameter of viable tumors (p = 0.0022), the target tumor long-axis diameter (p = 0.0019), and the target tumor area (p = 0.0007) were significantly larger in the cTACE/iBT group compared to the iBT group. Furthermore, prior treatment of non-target tumors differed significantly between groups (p = 0.0028), reflecting differences in prior disease management. Details regarding patient and treatment characteristics are provided in Tables 1 and 2. Laboratory parameters were presented in Supplementary Table S1.

### Comparison of baseline serum cytokines in the iBT and cTACE/iBT group

At baseline, IL-6 (5.67 pg/mL vs. 2.73 pg/mL; *p =* 0.0322) and IL-4 (0.036 pg/mL vs. 0.023 pg/mL; *p =* 0.0364) were significantly higher in the iBT group compared with the cTACE/iBT group. No significant differences between the two groups were observed for other cytokines or angiogenic factors (Supplementary Table S5).

### Comparison of pre-treatment vs. post-cTACE serum cytokines

**Figure 3:**
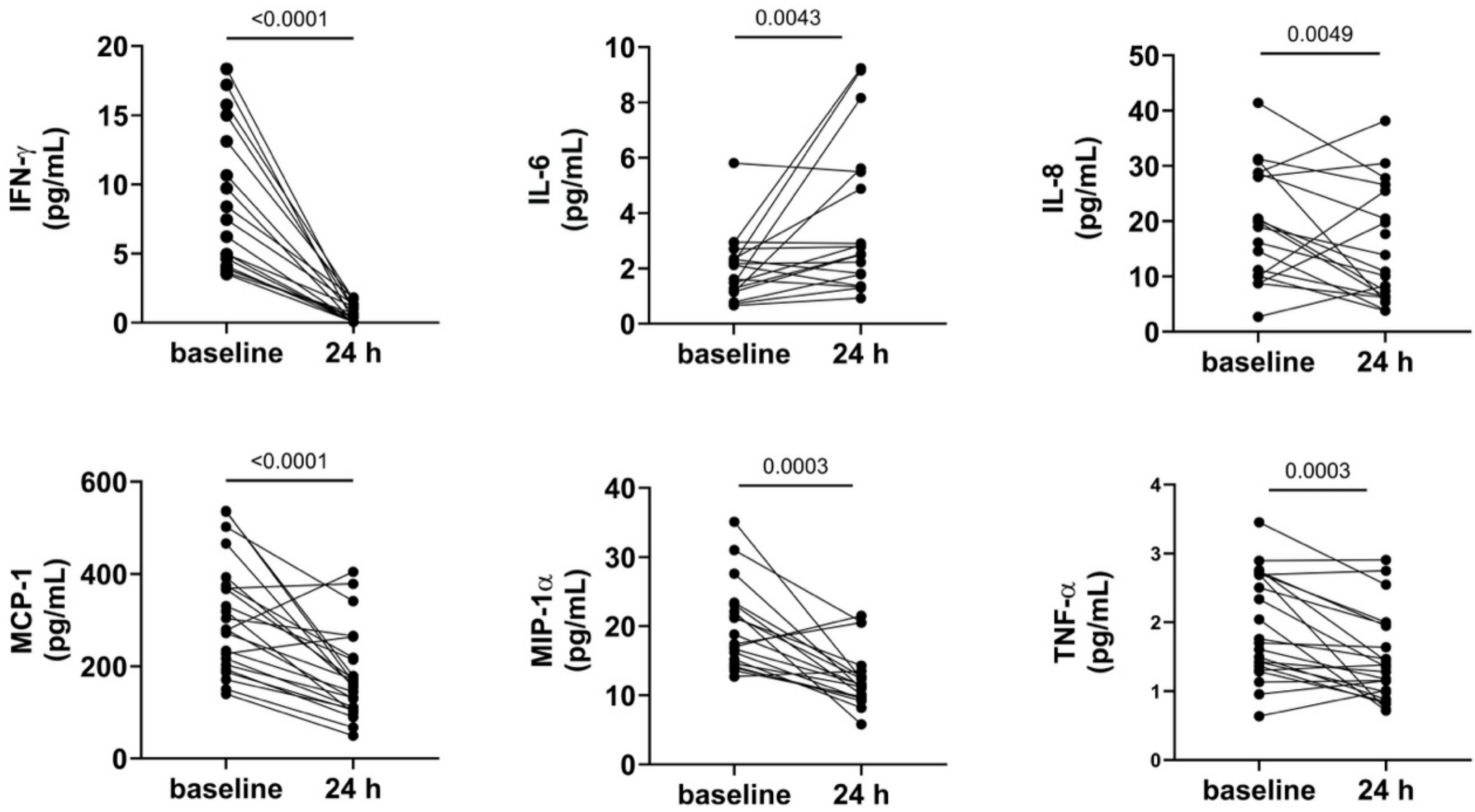
** Shows serum cytokine shifts post-TACE.** Legend: Serum cytokine levels measured at baseline and 24 hours after cTACE are shown. IFN-γ, MCP-1, MIP-1α, and TNF-α levels decreased significantly following cTACE (p < 0.0001 for IFN-γ and MCP-1; p = 0.0003 for MIP-1α and TNF-α). In contrast, IL-6 levels increased significantly (p = 0.0043). IL-8 levels also changed significantly between baseline and post-cTACE (p = 0.0049). Outliers were excluded from visualization; however, p-values were calculated using the complete dataset.

Significant decreases post-cTACE were observed for IFN-γ (14.68 pg/mL vs. 3.01 pg/mL; p < 0.0001), MCP-1 (306.6 pg/mL vs. 179.4 pg/mL; p < 0.0001), TNF-α (2.27 pg/mL vs. 1.44 pg/mL; p = 0.0003), MIP-1α (3.65 pg/mL vs. 2.21 pg/mL; p = 0.0003), IL-17 (3.65 pg/mL vs. 2.21 pg/mL; p = 0.0049), and IL-8 (40.75 pg/mL vs. 18.22 pg/mL; p = 0.0049). Conversely, IL-6 significantly increased post-cTACE (2.73 pg/mL vs. 8.08 pg/mL; p = 0.0043), (Figure 3). Anti-inflammatory cytokines such as IL-4 (0.023 pg/mL vs. 0.010 pg/mL; p = 0.0089) and IL-5 (0.776 pg/mL vs. 0.431 pg/mL; p = 0.0006) also significantly decreased post-treatment. However, proangiogenic factors VEGF-A and bFGF did not show significant changes. Detailed numerical values for all cytokines are provided in Supplementary Table S6.

### Comparison of TIF cytokines in untreated vs. post-cTACE tumors

Cytokine profiles in TIF were compared between untreated tumors (iBT group) and post-cTACE tumors (cTACE/iBT group). IL-17 was significantly higher in untreated tumors (30.94 pg/mL vs. 9.77 pg/mL, *p =* 0.0381). Other pro-inflammatory cytokines (IL-1β, IL-2, IL-6, IL-8, TNF-α, and MIP-1α) and anti-inflammatory cytokines (IL-4, IL-10, and IL-13) were also higher in untreated tumors, but without statistical significance. All numerical data is presented in Supplementary Table S7 and visualized in Supplementary Figure S2.

### Association of serum and TIF cytokines with imaging-based tumor response in the cTACE/iBT group

Detailed response distributions for each criterion are provided in Supplementary Tables S2-S4. No significant differences were found in baseline serum or TIF cytokine levels between responders (n = 5) and non-responders (n = 17) according to RECIST. When stratified by mRECIST, responders (n = 15) showed higher baseline serum MCP-1 compared to non-responders (n = 6), but without statistical significance (p = 0.1537). Post-cTACE, serum levels of bFGF were significantly higher in non-responders (p = 0.0449), while TIF IL-6 was higher in responders (p = 0.1781), but without statistical significance. Full data is provided in Supplementary Tables S8a-c and S9a-c.

### Association of serum and TIF cytokines with imaging-based tumor response in the iBT group

No significant differences in baseline serum cytokine levels were observed between responders (n = 8) and non-responders (n = 15) according to RECIST. Responders had significantly higher TIF IL-8 (p = 0.0103), and VEGF-A (p = 0.0185) levels compared to non-responders. When stratified by mRECIST, baseline serum IFN-γ (p = 0.0193), IL-4 (p = 0.0121), and bFGF (p = 0.0114) levels differed significantly between responders (n = 17) and non-responders (n = 5), but no significant differences were found in corresponding TIF levels. Full data is provided in Supplementary Tables S8d-e and S9d-e.

### Association of serum and TIF cytokines with lipiodol patterns

Post-cTACE, serum levels of IL-1β were significantly lower in patients with homogeneous Lipiodol deposition compared to the heterogeneous group (0.02 ± 0.007 vs. 0.14 ± 0.05 pg/mL, p = 0.0312). Similarly, serum VEGF-A levels were also lower in the homogeneous group compared to the heterogeneous group (313.5 ± 127.3 pg/mL vs. 614 ± 86.2 p = 0.0318). In contrast, no statistically significant differences were found in pre-treatment serum or TIF cytokines when stratified according to Lipiodol deposition. Full data were provided in Supplementary Table S10.

**Figure 4:**
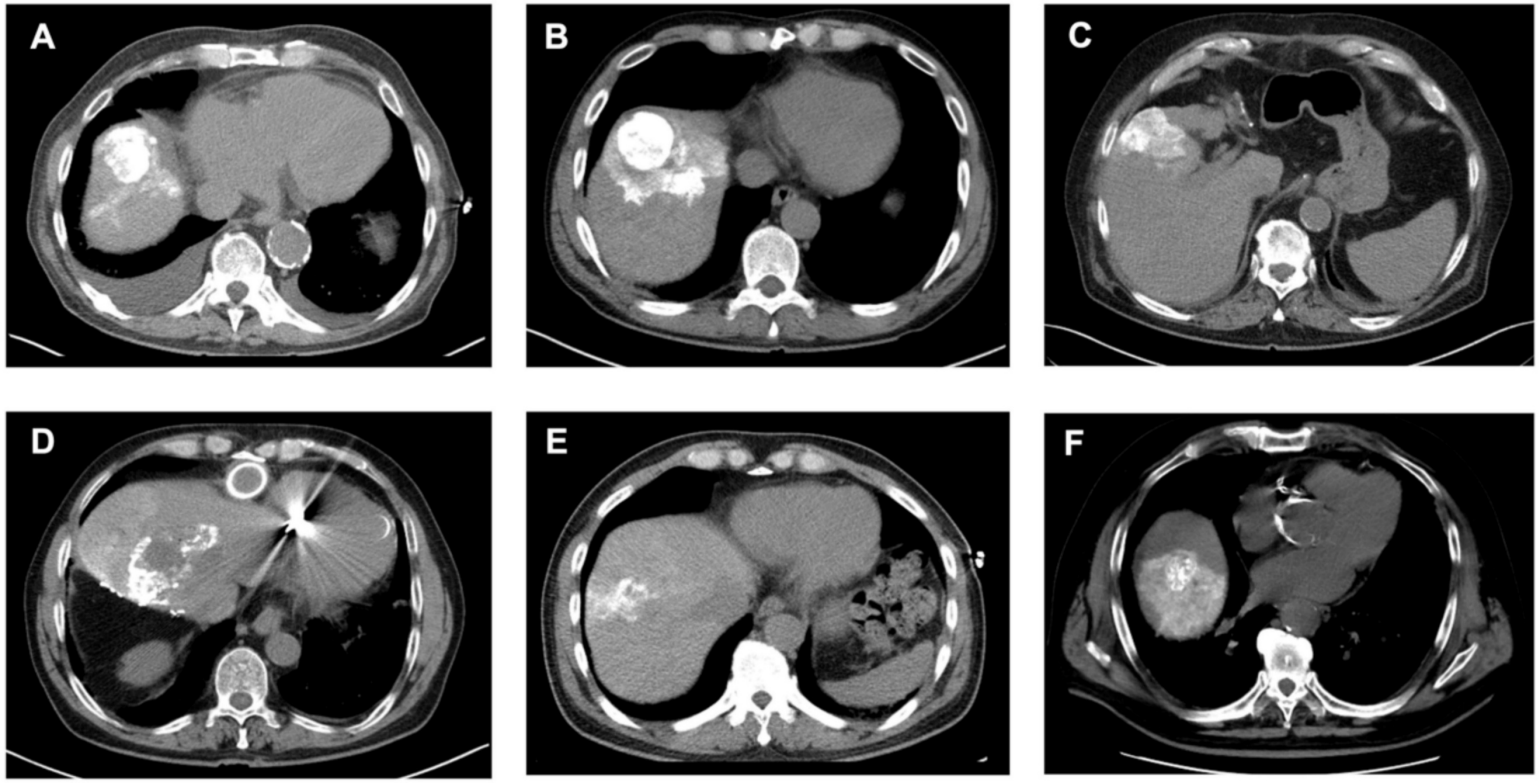
** Analysis of Lipiodol Deposition and Distribution Patterns post-cTACE.** Legend: CT scans obtained 24 hours after cTACE show different patterns of Lipiodol deposition within target tumors. Homogeneous deposition (A-C) was defined as ≥85% of the tumor volume containing medium or high-density Lipiodol. Heterogeneous deposition (D-F) was defined as <85% intratumoral Lipiodol uptake, characterized by patchy, irregular, or incomplete filling of the tumor. Lipiodol distribution was classified as confined to the tumor, without visible extension into the surrounding liver parenchyma (i.e., A), extending into one adjacent liver segment, without extension into additional segments (i.e., B, E, F), or extending beyond the tumor-bearing segment into at least one adjacent liver segment (i.e., D).

**Figure 5:**
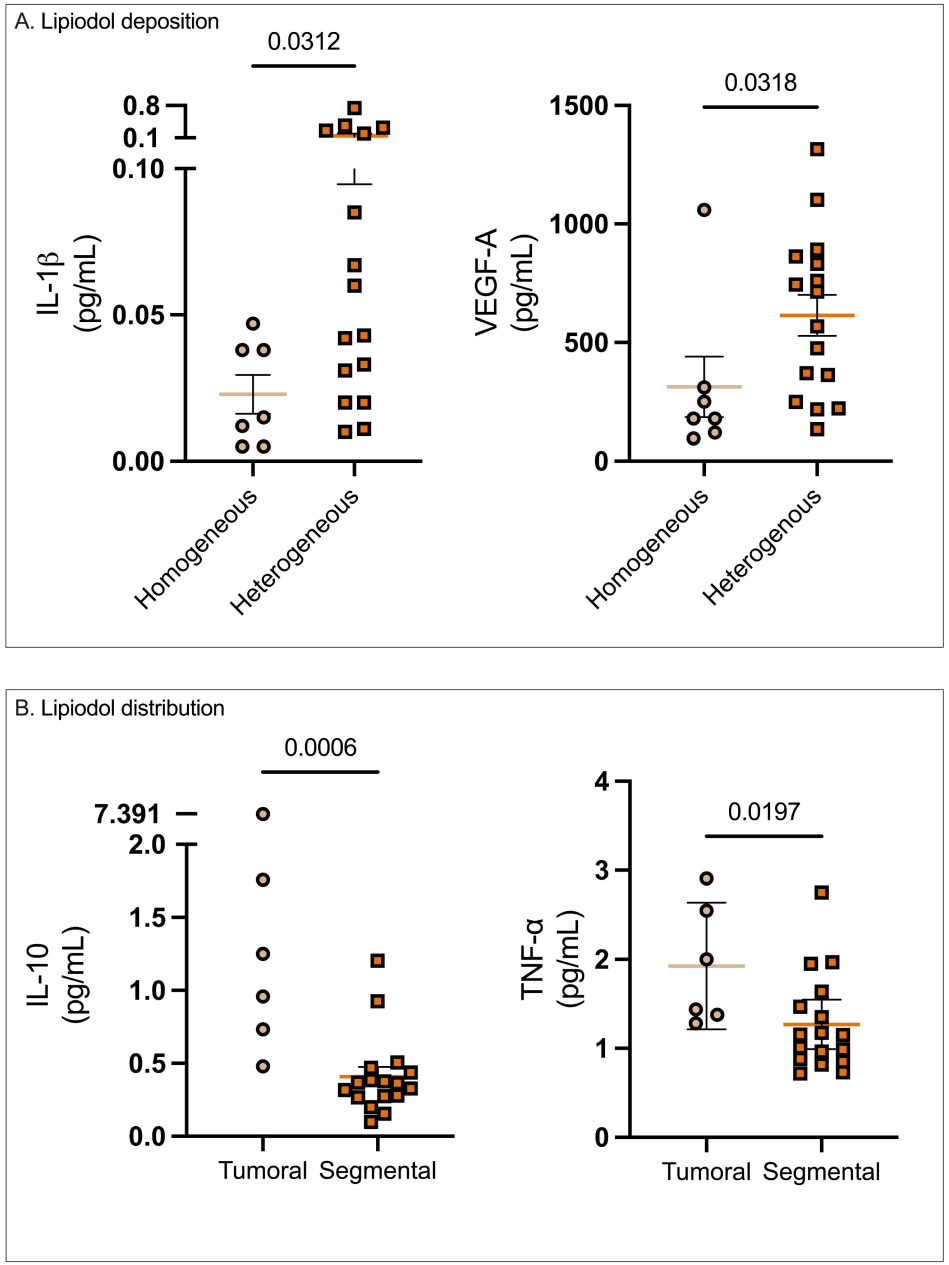
** Association of Lipiodol deposition and distribution patterns with post-cTACE serum cytokine levels.** Legend: (A) Patients with homogeneous Lipiodol deposition exhibited significantly lower post-cTACE serum IL-1β (p = 0.0312) and VEGF-A (p = 0.0318) compared to those with heterogeneous deposition. (B) Stratification by Lipiodol distribution pattern revealed significantly higher post-cTACE serum IL-10 (p = 0.0006) and TNF-α (p = 0.0197) levels in patients with tumoral-only deposition compared to those with segmental distribution.

However, when patients were stratified according to Lipiodol distribution, baseline serum IL-6 was significantly elevated in patients with tumoral compared to the segmental Lipiodol distribution (3.75 ± 0.73 pg/mL vs. 2.37 ± 0.65 pg/mL, p = 0.0033). In post-cTACE serum, IL-10, IL-4, TNF-α, and IL-5 were significantly higher in the tumoral group compared to the “segmental” group (IL-10: 2.1 ± 1.07 pg/mL vs. 0.41 ± 0.07 pg/mL, p = 0.0006; IL-4: 0.016 ± 0.003 pg/mL vs. 0.008 ± 0.004 pg/mL, p = 0.0018; TNF-α: 1.93 ± 0.28 pg/mL vs. 1.27 ± 0.13 pg/mL, p = 0.0197; IL-5: 0.97 ± 0.64 pg/mL vs. 0.24 ± 0.06 pg/mL, p = 0.0375). Post-cTACE TIF cytokine analysis revealed no statistically significant differences between patients with tumoral and segmental Lipiodol distribution, respectively. Full data is provided in Supplementary Table S11. Representative imaging examples illustrating Lipiodol deposition and distribution patterns are shown in Figure 4. The associations between Lipiodol deposition and distribution patterns and post-cTACE serum cytokine levels are summarized in Figure 5.

### Survival analysis

The mean follow-up duration for the entire cohort was 14.3 months (median: 15.5 months, Interquartile Range (IQR): 11.1-17.3 months). Overall, 19 patients (41.3%) died during follow-up. The median OS was 20.4 months, with estimated survival probabilities of 86.5% at 6 months and 77.4% at 12 months, respectively. Median OS was 20.4 months in the iBT group and 16.7 months in the cTACE/iBT group (p = 0.5036; Figure S3).

**Figure 6:**
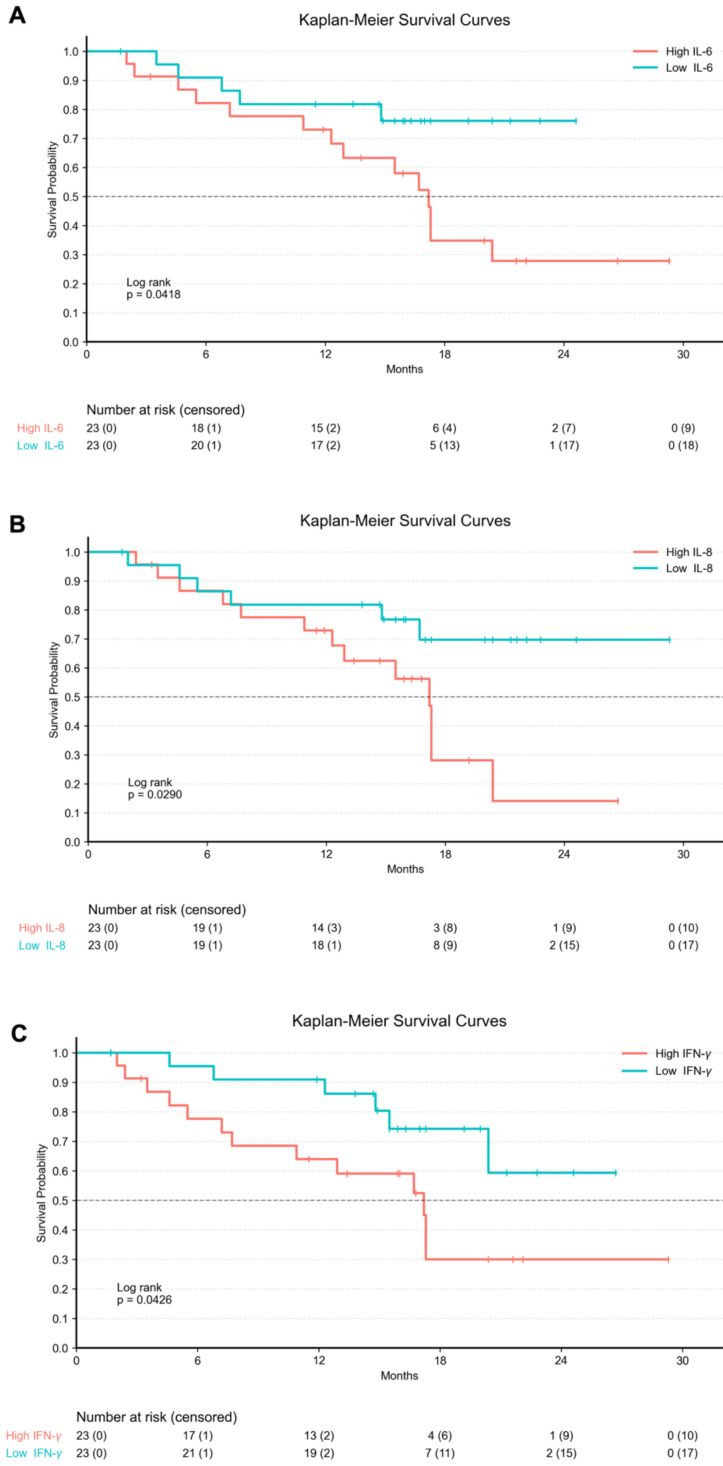
** Kaplan-Meier Curves for baseline serum cytokine levels in the entire cohort**. Legend: Kaplan-Meier survival curves illustrating overall survival stratified by baseline serum cytokine levels in the entire cohort (n = 46). Tick marks indicate censored observations. Cytokine concentrations were dichotomized into high and low groups based on the median baseline value of each marker. (A) Patients with high baseline serum IL-6 levels exhibited significantly shorter OS compared to those with low IL-6 levels (log-rank p = 0.0418). (B) Elevated baseline serum IL-8 levels were significantly associated with worse OS (log-rank p = 0.0290). (C) Higher baseline serum IFN-γ levels were associated with significantly improved OS compared to lower levels (log-rank p = 0.0426).

In addition, Kaplan-Meier survival analyses were performed by stratifying the entire cohort according to baseline serum cytokine levels. Patients were dichotomized into high and low groups based on the median cytokine concentration. Higher baseline serum IL-6 levels were associated with significantly shorter OS compared to lower IL-6 levels (p = 0.0418). Similarly, elevated baseline serum IL-8 was significantly associated with worse OS (p = 0.0290). In contrast, patients with higher baseline serum IFN-γ levels showed significantly improved OS compared with those with lower IFN-γ levels (p = 0.0426; Figure 6).

In univariate analysis of OS in the entire cohort, older age, larger target tumor size (long-axis diameter ≥30 mm), and impaired liver function as reflected by elevated AST and INR were associated with poorer survival. Among baseline serum cytokines, higher levels of IFN-γ, IL-1β, IL-6, IL-8, IL-17, and VEGF-A were associated with reduced OS in univariate analyses. In addition, a higher venous-phase tumor-to-liver contrast ratio on MRI was associated with improved OS (Supplementary Table S12).

In multivariate Cox regression analyses of the entire cohort, however, none of the variables were independently associated with OS at a significance level of p < 0.05 (Supplementary Table S13).

In the iBT cohort, univariate Cox regression analysis revealed elevated pre-treatment serum IL-8, MCP-1, and IL-13 levels, as well as higher TIF IL-1β and IL-8 levels as predictors of poorer OS, whereas higher TIF IL-5 and IFN-γ levels were associated with improved outcomes. In addition, older age and elevated AST levels were associated with poorer OS (Supplementary Table S14).

In the iBT cohort, multivariate Cox regression analyses were performed using three different models. In Model 1, which included age, selected serum cytokines, and TIF cytokines, higher IL-1β (TIF) levels were independently associated with poorer OS. In Model 2, including age and selected serum cytokines, older age and higher IL-13 (serum) levels were independently associated with reduced OS. In Model 3, combining age, IL-13 (serum), and IL-1β (TIF), both older age and elevated IL-13 (serum) and IL-1β (TIF) levels remained independently associated with OS (Supplementary Table S15). In the cTACE/iBT cohort, no cytokine parameter showed a significant association with OS.

## Discussion

This prospective study revealed distinct systemic and local immune alterations in HCC treated with cTACE with prognostic relevance. Systemically, cTACE triggered a pronounced acute inflammatory shift characterized by a significant rise in serum IL-6 accompanied by suppression of multiple pro-inflammatory cytokines, including IFN-γ, MCP-1, TNF-α, IL-17, and IL-8. In contrast, local immune changes within the TIF were more subtle, although untreated tumors exhibited significantly higher IL-17 levels, suggesting a pre-existing immunosuppressive microenvironment 40,41. Importantly, baseline systemic inflammation was strongly associated with outcome, as elevated serum IL-6, IL-8, and IFN-γ predicted poorer OS, whereas higher intratumoral IFN-γ levels were linked to improved prognosis, underscoring the paradoxical function of acute vs. chronic IFN-γ exposure. Furthermore, distinct cytokine patterns correlated with treatment response and Lipiodol deposition patterns, underscoring the complementary value of integrating systemic, local, and imaging biomarkers for biologically informed patient stratification.

IL-6 functions as a mediator of tissue repair, and its post-TACE elevation may reflect an early inflammatory response. However, persistent IL-6 elevation may foster chronic inflammation and immune evasion, contributing to tumor progression 15. In our study, elevated baseline serum levels of IL-6 and IL-8 were strong predictors of poor prognosis, consistent with their established roles in immune suppression and therapy resistance 42. Additionally, the increase of IL-6 post-cTACE may reflect a compensatory inflammatory surge, aligning with previous studies linking IL-6 to tumor progression 14,43. Together with the significant decrease of pro-inflammatory serum markers within 24 hours post-cTACE, this finding suggests a transient suppression of T-cell-mediated tumor immunity, potentially facilitating tumor immune escape. While cellular immune profiling was not performed in this study, prior studies report lymphodepletion within 24 hours post-cTACE, which is congruent with our observation of early systemic immune suppression 44.

This study employed a novel approach by analyzing TIF extracted directly from fresh tumor biopsies to characterize the local immune environment in HCC. Because TIF contains locally secreted signaling molecules with distinct spatial context compared with serum, the combined analysis of local and systemic compartments enables the detection of cTACE-induced immune alterations that may be obscured in circulation. Nevertheless, TIF analysis remains an emerging technique with inherent variability due to varying biopsy composition and possible blood micro-contamination, underscoring both the potential and the analytical challenges of interpreting TIF cytokine data 31. TIF has previously been utilized in various solid tumors to identify biomarkers associated with disease progression and therapeutic response 30,32. However, its application to assess immune alterations following cTACE in HCC has not been reported previously. Within TIF, elevated IL-8 and VEGF-A in responders to iBT indicated that these cytokines may play a role in mediating tumor vascularization and therapeutic susceptibility. IL-8, through neutrophil recruitment and angiogenic activity, emerged as a particularly compelling target for therapeutic intervention, which has been explored in both preclinical tumor models and early-phase clinical trials evaluating IL-8/CXCR2 blockade in melanoma, prostate, and renal cell carcinoma 43,45,46. Conversely, non-responders exhibited increased bFGF, a known driver of angiogenesis and resistance to therapy. Meanwhile, IFN-γ, a key anti-tumor cytokine in acute inflammation, was lower in non-responders compared to responders, and was associated with poorer survival, suggesting it as a relevant biomarker for an immune-suppressive TME 47.

These findings indicate that cytokine profiling could be implemented during pre-procedural blood work for baseline risk stratification and within 24 hours post-cTACE to identify patients at risk for poor response or unfavorable immune modulation, alongside standard laboratory testing and imaging. Pending, external validation in independent, multicenter cohorts, this information could support early selection of patients for cTACE-ICI combinations or prompt individual treatment escalation rather than waiting for delayed radiologic response assessment.

Lipiodol, an iodized poppy seed oil, is a radiopaque contrast agent and emulsifier utilized during cTACE for selective tumor embolization and drug delivery. It functions as both a carrier for chemotherapeutic agents and a non-invasive imaging biomarker for treatment response 48,49. Studies suggest that Lipiodol retention within the tumor can reflect both vascular architecture and tumor viability, making it a valuable tool for identifying patients who may benefit from additional systemic therapies, particularly with anti-angiogenic agents or immune checkpoint inhibitors 50-52. In this study, patterns of Lipiodol deposition emerged as significant modifiers of the immune response to cTACE. Specifically, patients exhibiting homogeneous Lipiodol tumoral deposition showed substantially lower serum IL-1β and VEGF-A levels and prolonged OS, possibly due to a more effective tumor embolization, more tumor-associated antigen release, and reduced hypoxia-driven angiogenesis.

Tumor size was an independent predictor of poor OS, in line with previously published clinical data. Elevated baseline serum IL-8, IL-17, and MCP-1 were associated with poorer OS, supporting previous findings that link these cytokines to angiogenesis, inflammation, and tumor progression 45,46,53. Among the cytokines associated with survival, IL-17 and MCP-1 have already been reported to shape immunosuppressive and pro-angiogenic TME in HCC, contributing to tumor progression and recurrence post-TACE 40,41. Elevated MCP-1, a key chemokine for monocyte recruitment, may indicate the infiltration of tumor-associated macrophages (TAMs) with an immunosuppressive M2 phenotype, fostering tumor regrowth and poor survival 54. Their post-cTACE elevation suggests that treatment-induced hypoxia may amplify these pro-tumorigenic pathways and thereby promote immune evasion and limiting the long-term efficacy.

This study has several limitations. Although prior work suggests that measurable immune shifts can occur as early as 24 hours after treatment, the absence of longitudinal sampling limits insights into the kinetics and duration of immune modulation 55. Additionally, the follow-up period may not fully capture long-term survival benefits in this BCLC A/B-dominant cohort, and limits events in survival analysis. While cytokine profiling provided valuable insights into the humoral immune response, additional methods such as flow cytometry would be needed to determine specific cellular responses induced by LRT. However, we have previously investigated and published the cellular immune responses post-cTACE in a similar patient cohort for comparison 44. Furthermore, histopathologic immune cell infiltration was not assessed in this study, limiting spatial and morphological context. However, biopsies were obtained under CT guidance, allowing for imaging confirmation of intra-tumoral sampling. However, in HCC—typically classified as a “cold” tumor—immune cells are known to accumulate preferentially in the peritumoral stroma rather than in the tumor core. This distribution may have restricted the detection of relevant immune cell populations in the analyzed biopsy material, whereas secreted cytokines in the TIF remain measurable 56. Group imbalances and patient heterogeneity in liver function and tumor burden may influence cytokine responses but have been accounted for using multivariate regression modeling. Additionally, the cohort includes consecutively recruited patients, strengthening data robustness and translational relevance. While this study identifies cytokine changes induced by cTACE, response to therapy and survival measures account for combined cTACE/iBT. Lastly, Lipiodol patterns were assessed qualitatively rather than using a quantitative approach. However, the applied technique is well-documented and easily implementable, making it a promising tool for integration into post-cTACE patient assessment 37,39.

In conclusion, this prospective study demonstrates that cTACE induces systemic and local immune alterations in HCC, captured by cytokine profiling in peripheral blood and tumor biopsies. Elevated IL-6, IL-8, and IFN-γ in circulation predicted poor OS, while intratumoral IFN-γ signaled enhanced T-cell activity and improved survival. Conversely, high baseline MCP-1 and IL-17 correlated with immunosuppressive myeloid recruitment and resistance to therapy. Imaging biomarkers complemented these findings, with homogeneous Lipiodol deposition linked to reduced VEGF-A levels and improved survival. Together, these data highlight the value of integrating liquid biopsy, tumoral immune profiling, and imaging to personalize locoregional treatment strategies in HCC.

## Supplementary Material

Supplementary methods, figures and tables.

## Figures and Tables

**Table 1:** Baseline Patients and Disease Characteristics of the Cohort. Legend: Baseline demographic, clinical, and disease characteristics of the study cohort. Data were presented for the entire cohort and stratified by treatment group (iBT vs. cTACE/iBT). Values were given as mean ± standard deviation (SD) for continuous variables and number (percentage) for categorical variables Between-group comparisons were performed using the Mann-Whitney U test for continuous variables and Fisher's exact test for categorical variables. Statistically significant differences were indicated by asterisks (*p < 0.05, **p < 0.01, ***p < 0.001).

Patient Characteristics	Entire Cohort (n = 46)	iBT (n = 23)	cTACE/iBT (n = 23)	P Value
Gender, n (%)				
m	36 (78%)	14 (61%)	22 (96%)	**0.0098
f	10 (22%)	9 (39%)	1 (4%)	
Age (years), mean ± SD	71.4 ± 9.5	69.4 ± 10.0	73.2 ± 8.7	0.1285
Disease Characteristics				
Liver Cirrhosis, n (%)	44 (96%)	22 (96%)	22 (96%)	> 0.9999
Etiology of cirrhosis, n (%)				
				0.1274
NASH	11 (25%)	4 (18%)	7 (32%)	
Alcohol	11 (25%)	7 (32%)	4 (18%)	
Hepatitis C	7 (16%)	6 (27%)	1 (4%)	
Hepatitis B	3 (7%)	0	3 (14%)	
Other*	4 (9%)	2 (9%)	2 (9%)	
Unknown	8 (18%)	3 (14%)	5 (23%)	
Ascites, n (%)	21 (46%)	11 (48%)	10 (43%)	> 0.9999
Splenomegaly, n (%)	21 (46%)	13 (57%)	8 (35%)	0.2362
Child-Pugh, n (%)				0.2333
A	43 (93%)	23 (100%)	20 (86%)	
B	3 (7%)	0	3 (13%)	
MELD Score, mean ± SD	9 ± 3.1	8.7 ± 2.8	9.3 ± 3.5	0.7891

*Others: alcohol+NASH (n = 1), HCV+NASH, (n = 1), primary biliary cholangitis (n = 1), Alpha-1 antitrypsin deficiency (n = 1) m: male, f: female, NASH: Non-alcohol related steatohepatitis, MELD: Model of end-stage liver disease, was not available for 1 patient in entire cohort, N: number of patients, SD: standard deviation.

**Table 2:** ** Tumor and Target Tumor Characteristics.** Legend: Data were presented for the entire cohort and stratified by treatment group (iBT vs. cTACE/iBT). Values were shown as mean ± standard deviation (SD) for continuous variables and number (percentage) for categorical variables. Between-group comparisons were performed using *the* Mann*-*Whitney U test for continuous variables and Fisher's exact test for categorical variables*.* Statistically significant differences were indicated by asterisks (*p < 0.05, **p < 0.01, ***p < 0.001).

Tumor Characteristics	Entire Cohort (n = 46)	iBT (n = 23)	cTACE/iBT (n = 23)	P Value
Number of viable tumors, n (%)				0.43630
Single	29 (63%)	15 (65%)	14 (61%)	
Multiple (n = 2)	13 (28%)	5 (22%)	8 (35%)	
Multiple (n = 3)	4 (9%)	3 (13%)	1 (4%)	
Total diameter of viable tumors, (mm), mean ± SD	41.1 ± 23.6	30.9 ± 18.5	51.3 ± 24.1	**0.0022
AFP (µg/l), mean ± SD	42.4 ± 141.4	30.5 ± 51.5	66.1 ± 198.4	0.5737
BCLC Stage				*0.0012
A	31 (67%)	21 (91%)	10 (43%)	
B	15 (33%)	2 (9%)	13 (57%)	
Prior treatments of non-target tumors				**0.0028
Locoregional treatment	20 (47%)	17 (74%)	4 (17%)	
Surgical treatment	4 (9%)	0	4 (17%)	
Systemic treatment	2 (4%)	1 (4%)	1 (4%)	
Target Tumor Characteristics				
Long axis (mm) mean ± SD	33.7 ± 22.6	25.0 ± 19.9	42.3 ± 23.1	**0.0019
Area (mm²) mean ± SD	1332.7 ± 1944.9	757.1 ± 1705.7	1908.3 ± 2033.5	***0.0007
LI-RADS Stage, n (%)				*0.0459
LR-4	8 (18%)	7 (32%)	1 (5%)	
LR-5	36 (82%)	15 (68%)	21 (95%)	

BCLC: Barcelona Clinic Liver Cancer staging system, AFP: alpha-fetoprotein, available for 30 patients in entire cohort, LI-RADS: The Liver Imaging Reporting and Data System, were not available for 2 patients.

## Data Availability

The datasets generated and/or analyzed during the current study are available from the corresponding author on reasonable request.

## Abbreviations

AFP: alpha-fetoprotein
AST: aspartate aminotransferase
BCLC: Barcelona Clinic Liver Cancer
bFGF: basic fibroblast growth factor
CI: confidence interval
cTACE: conventional transarterial chemoembolization
CT: computed tomography
ECOG: Eastern Cooperative Oncology Group
HCC: hepatocellular carcinoma
HR: hazard ratio
H&E: hematoxylin and eosin
ICI: immune checkpoint inhibitor
IFN-γ: interferon-gamma
IL: interleukin
iBT: interstitial brachytherapy
IQR: interquartile range
LI-RADS: Liver Imaging Reporting and Data System
LRT: locoregional therapy
MCP-1: monocyte chemoattractant protein-1
MELD: Model for End-Stage Liver Disease
MIP-1α: macrophage inflammatory protein-1 alpha
MRI: magnetic resonance imaging
mRECIST: modified Response Evaluation Criteria In Solid Tumors
MSD: Meso Scale Discovery
NASH: non-alcoholic steatohepatitis
OS: overall survival
RECIST: Response Evaluation Criteria In Solid Tumors
ROI: region of interest
SD: standard deviation
SST: serum separator tube
TACE: transarterial chemoembolization
TAM: tumor-associated macrophage
TIF: tumor interstitial fluid
TME: tumor microenvironment
TNF-α: tumor necrosis factor-alpha
VEGF: vascular endothelial growth factor
VEGF-A: vascular endothelial growth factor A

## References

[1] Bray F, Laversanne M, Sung H, Ferlay J, Siegel RL, Soerjomataram (2024). Global cancer statistics 2022: GLOBOCAN estimates of incidence and mortality worldwide for 36 cancers in 185 countries. CA Cancer J Clin.

[2] Guo J, Wang S, Han Y, Jia Z, Wang R (2021). Effects of transarterial chemoembolization on the immunological function of patients with hepatocellular carcinoma. Oncol Lett.

[3] Lemieux S, Buies A, F (2021). Turgeon A, Hallet J, Daigle G, Côté F, et al. Effect of Yttrium-90 transarterial radioembolization in patients with non-surgical hepatocellular carcinoma: A systematic review and meta-analysis. Huang Y-H, Ed. PLOS ONE.

[4] Renzulli M, Peta G, Vasuri F, Marasco G, Caretti D, Bartalena L (2021). Standardization of conventional chemoembolization for hepatocellular carcinoma. Ann Hepatol.

[5] Sieghart W, Hucke F, Pinter M, Graziadei I, Vogel W, Müller C (2013). The ART of decision making: retreatment with transarterial chemoembolization in patients with hepatocellular carcinoma. Hepatology.

[6] Hucke F, Pinter M, Graziadei I, Bota S, Vogel W, Müller C (2014). How to STATE suitability and START transarterial chemoembolization in patients with intermediate stage hepatocellular carcinoma. J Hepatol.

[7] Tischfield DJ, Gurevich A, Johnson O, Gatmaytan I, Nadolski GJ, Soulen MC (2022). Transarterial Embolization Modulates the Immune Response within Target and Nontarget Hepatocellular Carcinomas in a Rat Model. Radiology.

[8] Lencioni R, Kudo M, Malagari K, Vogel A, Castera L, Sangro B (2024). EMERALD-1: A phase 3, randomized, placebo-controlled study of transarterial chemoembolization (TACE) combined with durvalumab and bevacizumab therapy in patients with unresectable hepatocellular carcinoma. J Clin Oncol.

[9] Kudo M, Ren Z, Guo Y, Han G, Lin H, Zheng J (2025). Transarterial chemoembolisation combined with lenvatinib plus pembrolizumab versus dual placebo for unresectable, non-metastatic hepatocellular carcinoma (LEAP-012): a multicentre, randomised, double-blind, phase 3 study. The Lancet.

[10] Abou-Alfa GK, Fan J, Heo J, Arai Y, Erinjeri JP, Kuhl CK (2022). 727TiP A randomised phase III study of tremelimumab (T) plus durvalumab (D) with or without lenvatinib combined with concurrent transarterial chemoembolisation (TACE) versus TACE alone in patients (pts) with locoregional hepatocellular carcinoma (HCC): EMERALD-3. Ann Oncol.

[11] Sangro B, Kudo M, Erinjeri JP, Qin S, Ren Z, Chan SL Durvalumab with or without bevacizumab with transarterial chemoembolisation in hepatocellular carcinoma (EMERALD-1): a multiregional, randomised, double-blind, placebo-controlled, phase 3 study. Lancet. 2025.

[12] Quail DF, Joyce JA (2013). Microenvironmental regulation of tumor progression and metastasis. Nat Med.

[13] Kurebayashi Y, Ojima H, Tsujikawa H, Kubota N, Maehara J, Abe Y (2018). Landscape of immune microenvironment in hepatocellular carcinoma and its additional impact on histological and molecular classification. Hepatology.

[14] Loosen SH, Schulze-Hagen M, Leyh C, Benz F, Vucur M, Kuhl C (2018). IL-6 and IL-8 Serum Levels Predict Tumor Response and Overall Survival after TACE for Primary and Secondary Hepatic Malignancies. Int J Mol Sci.

[15] Taniguchi K, Karin M (2014). IL-6 and related cytokines as the critical lynchpins between inflammation and cancer. Semin Immunol.

[16] Sansone P, Bromberg J (2012). Targeting the Interleukin-6/Jak/Stat Pathway in Human Malignancies. J Clin Oncol.

[17] Wang T, Niu G, Kortylewski M, Burdelya L, Shain K, Zhang S (2004). Regulation of the innate and adaptive immune responses by Stat-3 signaling in tumor cells. Nat Med.

[18] Lee C, Cheung ST (2019). STAT3: An Emerging Therapeutic Target for Hepatocellular Carcinoma. Cancers.

[19] Lee YS, Choi I, Ning Y, Kim NY, Khatchadourian V, Yang D (2012). Interleukin-8 and its receptor CXCR2 in the tumour microenvironment promote colon cancer growth, progression and metastasis. Br J Cancer.

[20] Dwyer J, Hebda JK, Le Guelte A, Galan-Moya E-M, Smith SS, Azzi S (2012). Glioblastoma Cell-Secreted Interleukin-8 Induces Brain Endothelial Cell Permeability via CXCR2. Canoll P, Ed. PLoS ONE.

[21] Zhu YM, Webster SJ, Flower D, Woll PJ (2004). Interleukin-8/CXCL8 is a growth factor for human lung cancer cells. Br J Cancer.

[22] David J, Dominguez C, Hamilton D, Palena C (2016). The IL-8/IL-8R Axis: A Double Agent in Tumor Immune Resistance. Vaccines.

[23] Alfaro C, Sanmamed MF, Rodríguez-Ruiz ME, Teijeira Á, Oñate C, González Á (2017). Interleukin-8 in cancer pathogenesis, treatment and follow-up. Cancer Treat Rev.

[24] Benci JL, Johnson LR, Choa R, Xu Y, Qiu J, Zhou Z (2019). Opposing Functions of Interferon Coordinate Adaptive and Innate Immune Responses to Cancer Immune Checkpoint Blockade. Cell.

[25] Spranger S, Spaapen RM, Zha Y, Williams J, Meng Y, Ha TT (2013). Up-Regulation of PD-L1, IDO, and T_regs_ in the Melanoma Tumor Microenvironment Is Driven by CD8^+^ T Cells. Sci Transl Med.

[26] Melero I, Yau T, Kang Y-K, Kim T-Y, Santoro A, Sangro B (2024). Nivolumab plus ipilimumab combination therapy in patients with advanced hepatocellular carcinoma previously treated with sorafenib: 5-year results from CheckMate 040. Ann Oncol.

[27] Mo X, Zhang H, Preston S, Martin K, Zhou B, Vadalia N (2018). Interferon-γ Signaling in Melanocytes and Melanoma Cells Regulates Expression of CTLA-4. Cancer Res.

[28] Stojanovic A, Fiegler N, Brunner-Weinzierl M, Cerwenka A (2014). CTLA-4 Is Expressed by Activated Mouse NK Cells and Inhibits NK Cell IFN-γ Production in Response to Mature Dendritic Cells. J Immunol.

[29] Stylianopoulos T, Munn LL, Jain RK (2018). Reengineering the Physical Microenvironment of Tumors to Improve Drug Delivery and Efficacy: From Mathematical Modeling to Bench to Bedside. Trends Cancer.

[30] Wagner M, Wiig H (2015). Tumor Interstitial Fluid Formation, Characterization, and Clinical Implications. Front Oncol.

[31] Apiz Saab JJ, Muir A (2023). Tumor interstitial fluid analysis enables the study of microenvironment-cell interactions in cancers. Curr Opin Biotechnol.

[32] Celis JE, Gromov P, Cabezón T, Moreira JMA, Ambartsumian N, Sandelin K (2004). Proteomic Characterization of the Interstitial Fluid Perfusing the Breast Tumor Microenvironment. Mol Cell Proteomics.

[33] Sun W, Ma J, Wu S, Yang D, Yan Y, Liu K (2010). Characterization of the Liver Tissue Interstitial Fluid (TIF) Proteome Indicates Potential for Application in Liver Disease Biomarker Discovery. J Proteome Res.

[34] Ricke J, Wust P, Stohlmann A, Beck A, Cho CH, Pech M (2004). CT-guided interstitial brachytherapy of liver malignancies alone or in combination with thermal ablation: phase I-II results of a novel technique. Int J Radiat Oncol.

[35] Bretschneider T, Ricke J, Gebauer B, Streitparth F (2016). Image-guided high-dose-rate brachytherapy of malignancies in various inner organs - technique, indications, and perspectives. J Contemp Brachytherapy.

[36] Mohnike K, Wieners G, Schwartz F, Seidensticker M, Pech M, Ruehl R (2010). Computed Tomography-Guided High-Dose-Rate Brachytherapy in Hepatocellular Carcinoma: Safety, Efficacy, and Effect on Survival. Int J Radiat Oncol.

[37] Stark S, Wang C, Savic LJ, Letzen B, Schobert I, Miszczuk M (2020). Automated feature quantification of Lipiodol as imaging biomarker to predict therapeutic efficacy of conventional transarterial chemoembolization of liver cancer. Sci Rep.

[38] Dioguardi Burgio M, Sartoris R, Libotean C, Zappa M, Sibert A, Vilgrain V (2019). Lipiodol retention pattern after TACE for HCC is a predictor for local progression in lesions with complete response. Cancer Imaging.

[39] Savic LJ, Chapiro J, Funai E, Bousabarah K, Schobert IT, Isufi E (2021). Prospective study of Lipiodol distribution as an imaging marker for doxorubicin pharmacokinetics during conventional transarterial chemoembolization of liver malignancies. Eur Radiol.

[40] Numasaki M (2003). Interleukin-17 promotes angiogenesis and tumor growth. Blood.

[41] Harley ITW, Stankiewicz TE, Giles DA, Softic S, Flick LM, Cappelletti M (2014). IL-17 Signaling Accelerates the Progression of Nonalcoholic Fatty Liver Disease in Mice. Hepatology.

[42] Myojin Y, Kodama T, Sakamori R, Maesaka K, Matsumae T, Sawai Y (2022). Interleukin-6 Is a Circulating Prognostic Biomarker for Hepatocellular Carcinoma Patients Treated with Combined Immunotherapy. Cancers.

[43] Yi M, Li T, Niu M, Zhang H, Wu Y, Wu K (2024). Targeting cytokine and chemokine signaling pathways for cancer therapy. Signal Transduct Target Ther.

[44] Schmidt R, Gebauer B, Akbari N, Roderburg C, Torsello GF, Fehrenbach U (2025). Immunologic effects of locoregional therapies for unresectable hepatocellular carcinoma. JHEP Rep.

[45] Teijeira A, Garasa S, Ochoa MC, Villalba M, Olivera I, Cirella A (2021). IL8, Neutrophils, and NETs in a Collusion against Cancer Immunity and Immunotherapy. Clin Cancer Res.

[46] Rizzo M, Varnier L, Pezzicoli G, Pirovano M, Cosmai L, Porta C (2022). IL-8 and its role as a potential biomarker of resistance to anti-angiogenic agents and immune checkpoint inhibitors in metastatic renal cell carcinoma. Front Oncol.

[47] Gao J, Shi LZ, Zhao H, Chen J, Xiong L, He Q (2016). Loss of IFN-γ Pathway Genes in Tumor Cells as a Mechanism of Resistance to Anti-CTLA-4 Therapy. Cell.

[48] Lencioni R, De Baere T, Soulen MC, Rilling WS, Geschwind JH (2016). Lipiodol transarterial chemoembolization for hepatocellular carcinoma: A systematic review of efficacy and safety data. Hepatology.

[49] Miyayama S, Matsui O (2016). Superselective Conventional Transarterial Chemoembolization for Hepatocellular Carcinoma: Rationale, Technique, and Outcome. J Vasc Interv Radiol.

[50] Golfieri R, Renzulli M, Mosconi C, Forlani L, Giampalma E, Piscaglia F (2013). Hepatocellular Carcinoma Responding to Superselective Transarterial Chemoembolization: An Issue of Nodule Dimension?. J Vasc Interv Radiol.

[51] Nezami N, Van Breugel JMM, Konstantinidis M, Chapiro J, Savic LJ, Miszczuk MA (2021). Lipiodol Deposition and Washout in Primary and Metastatic Liver Tumors After Chemoembolization. In Vivo.

[52] Vogl TJ, Naguib NNN, Nour-Eldin N-EA, Rao P, Emami AH, Zangos S (2009). Review on transarterial chemoembolization in hepatocellular carcinoma: Palliative, combined, neoadjuvant, bridging, and symptomatic indications. Eur J Radiol.

[53] You R, Jiang H, Xu Q, Yin G (2021). Preintervention MCP-1 serum levels as an early predictive marker of tumor response in patients with hepatocellular carcinoma undergoing transarterial chemoembolization. Transl Cancer Res.

[54] Tian Z, Hou X, Liu W, Han Z, Wei L (2019). Macrophages and hepatocellular carcinoma. Cell Biosci.

[55] Pinato DJ, Murray SM, Forner A, Kaneko T, Fessas P, Toniutto P (2021). Trans-arterial chemoembolization as a loco-regional inducer of immunogenic cell death in hepatocellular carcinoma: implications for immunotherapy. J Immunother Cancer.

[56] Jiang P, Gu S, Pan D, Fu J, Sahu A, Hu X (2018). Signatures of T cell dysfunction and exclusion predict cancer immunotherapy response. Nat Med.

