# Supplementary material for: Local and systemic cytokine signatures for outcome prediction after transarterial chemoembolization in hepatocellular carcinoma
Source: Theranostics. 2026 Apr 16;16(11):6099–112. doi: 10.7150/thno.130031 (PMC13142240; doi:10.7150/thno.130031)
Supplement: Supplementary file 1 — Supplementary methods, figures and tables. [file thnov16p6099s1.pdf]

Supplementary Methods

Locoregional Therapies

Conventional transarterial chemoembolization (cTACE): The cTACE procedures were performed under fluoroscopic guidance under (e.g., fentanyl [Janssen-Cilag, Neuss, Germany]; and midazolam [Hexal, Holzkirchen, Germany]) and local anesthesia with lidocaine (Xylocitin®, mibe GmbH, Brehna, Germany). The femoral artery was punctured, a sheath was placed in the common femoral artery, and a microcatheter (Progreat®, Terumo, Tokyo, Japan) was introduced into the common hepatic artery. Following that, the microcatheter was carefully inserted into the feeding artery of the tumor or positioned as close to the tumor as possible and inserted into the arteries with 5 cc of chemotherapy solution (50 mg doxorubicin [Adriblastina®, Pfizer, New York, NY, USA] in 2.5 cc and 10mg of mitomycin-C [Medac GmbH, Wedel, Germany] in 2.5 cc) mixed 1:2 with Lipiodol (approximately 10 cc; Guerbet, France) [1]. The solution was prepared following established protocols.

Interstitial brachytherapy (iBT): The iBT, using the afterloading technique, was performed according to the recommended guidelines described in previous studies [2,3]. Initially, a native spiral CT scan of the upper abdomen (Somatom, Siemens Healthineers, Erlangen, Germany) was acquired. This was followed by percutaneous CT-guided puncture and biopsy of the target tumor under sterile conditions, and insertion of the brachytherapy catheter. The procedure was carried out under analgesia (e.g., fentanyl and midazolam) and local anesthesia with lidocaine at the puncture site. Applicators used included a 17G trocar needle and a 6F AVANTI®+ sheath introducer (Cordis, Santa Clara, CA, USA), which were placed under CT guidance. After confirmation of proper placement, the catheters were fixed with sutures. A 6F angiographic sheath was used to deliver radiation directly to the target area. At the end of the procedure, a native spiral CT of the upper abdomen was performed to verify catheter positioning. For treatment planning, a contrast-enhanced CT (CECT) of the upper abdomen was acquired in the arterial phase (15 seconds after contrast injection), using a primary slice thickness of 0.625 mm and reconstructed to 5 mm slices. The patient was then transferred to the nuclear operating room. Imaging data were imported into the 3D radiation planning workstation, where, in collaboration with a medical physicist, an individualized irradiation plan was developed. Interstitial high-dose-rate (HDR) brachytherapy was delivered using Iridium-192 (GammaMedplus iX, Varian Medical Systems, Palo Alto, CA, USA) via the afterloading technique. After irradiation, the catheters were removed, and the needle tract was closed with a gelatin sponge (Gelfoam®, Pfizer, New York, NY, USA).

Imaging Protocol

Patients underwent baseline multiparametric MRI within 30 days prior to investigational therapy, except for two patients in the cTACE/iBT group who received baseline contrast-enhanced CT. For therapy planning and Lipiodol deposition assessment, contrast-enhanced CT imaging was performed for the iBT procedure. Follow-up imaging (MRI or CT) was conducted at 8–12 weeks post-LRT to evaluate tumor response.

Imaging acquisition: Prior to LRT, all patients received contrast-enhanced CT or MR imaging of the abdomen (baseline imaging). After the LRTs, patients were followed up within eight weeks and six months. Imaging was performed according to current institutional standards. Patients received non-contrast and contrast-enhanced multiphase T1-weighted images with a 1.5-Tesla scanner (Avanto and Aera; Siemens,

Erlangen, Germany), or a 3-Tesla-scanner (Skyra; all scanners from Siemens, Erlangen, Germany) with a phased-array torso coil. The MRI protocol was based on dynamic contrast-enhanced (CE) axial T1-weighted (T1WI) sequences: unenhanced, arterial (15 - 25 seconds after contrast administration), portal venous (70 - 90 seconds after contrast administration), and delayed/venous phases (3 minutes after contrast administration). It included axial diffusion-weighted imaging (DWI) with apparent diffusion coefficient (ADC) maps, axial T2-weighted (T2WI) single-shot fast spin-echo imaging, and axial hepatobiliary phase (HBP) imaging. HBP images were acquired before and 20 minutes after the bolus injection of 0.1 ml/kg body weight gadoxetic acid (Gd-EOB-DTPA, Primovist®; Bayer, Berlin, Germany). The multi-phasic contrast-enhanced CT was acquired with a multi-detector scanner (Siemens, Malvern, USA, PA) and included native scans, contrast-enhanced arterial, portal-venous, and venous phases. In patients undergoing cTACE, an additional unenhanced CT scan was acquired 24 hours post-procedure. Scan parameters included: collimation of 128 × 0.6 mm, tube voltage of 120 kVp, reference tube current of 230 mAs, pitch of 0.6, and a field of view (FOV) of 350 mm. Images were reconstructed using a medium-soft convolution kernel (B30f) at 3 mm slice thickness. All images were evaluated using both axial and coronal reformats.

Image Analysis

Baseline Imaging: Tumors were classified using LI-RADS criteria [4]. Signal intensity ratios between tumors and adjacent liver were calculated from different MRI phases (arterial, portal, venous, T2WI, DWI, hepatobiliary phases).

Signal intensities on baseline multiparametric MRI Scans: The Region of Interest (ROI) ratios for HCC patients were calculated to evaluate the tumor-to-liver contrast across multiple MRI phases. A single representative volume was analyzed for each target tumor. Circular ROIs of 10 mm^2 were manually placed within the tumor and normal liver tissue using images from the arterial, portal, venous, T2-weighted, diffusion-weighted (DWI), and hepatobiliary phases. Tumor ROIs were carefully positioned within enhancing regions, avoiding necrosis, hemorrhage, or artifacts, while liver ROIs were placed in homogenous parenchyma, away from vessels or bile ducts. Signal intensities (SI) were extracted for each ROI, and phase-specific ratios were calculated using the formula:

Ratio = Mean SI of Liver ROI/Mean SI of Tumor ROI

Response assessment: Target tumor response was assessed after each completed treatment on the baseline and 8-week follow-up imaging dataset according to the Response Evaluation Criteria In Solid Tumors version 1.1 (RECIST 1.1), the modified (m)RECIST criteria, and the Liver Imaging and Data Reporting System (LI-RADS). Two radiologists did the Treatment Response Algorithm (TRA) with three and eight years of experience in abdominal imaging. Images were viewed, and calculations were performed using Visage Picture Archiving and Communication Systems (PACS) client version 7 (Visage Imaging).

Two radiologists verified all measurements independently, and discrepancies were resolved by consensus. The extracted ratios and corresponding imaging data were securely recorded for statistical analysis.

Supplementary Tables

Laboratory Parameters	Entire Cohort (n = 46)	iBT (n = 23)	cTACE/iBT (n = 23)	P value
Total Bilirubin (mg/dL), mean ± SD	0.92 ± 0.48	0.85 ± 0.50	0.99 ± 0.45	0.1873
Albumin (g/L), mean ±SD	39.6 ± 4.8	39.6 ± 4.1	39.5 ± 5.4	0.9490
ALT (U/L), mean ±SD	38.6 ± 26.1	30.2 ± 15.6	46.6 ± 31.5	*0.0204
AST (U/L), mean ±SD	47.0 ± 18.7	41.5 ± 15.8	52.2 ± 20.2	*0.0446
GGT (U/L), mean ±SD	176.1 ± 171.4	176.5 ± 198.5	175.7 ± 144.1	0.5807
Hb (g/dL), mean ±SD	12.9 ± 2.2	12.6 ± 2.2	13.1 ± 2.3	0.5027
WBC (× 10^9/L), mean ±SD	5.52 ± 1.95	5.37 ± 1.92	5.69 ± 2.02	0.7236
Platelets count (× 10^9/L), mean ±SD	140.8 ± 62.9	141.7 ± 72.2	139.9 ± 53.7	0.7814
INR, mean ±SD	1.06 ± 0.16	1.07 ± 0.12	1.05 ± 0.19	0.5171
PTT (sec), mean ±SD	33.0 ± 5.1	33.0 ± 5.7	33.1 ± 4.6	0.9955
ALBI Score, mean ±SD	-2.61 ± 0.48	-2.65 ± 0.44	-2.57 ± 0.53	0.5648
ALBI Grade, n (%)				
Grade 1	23 (51%)	12 (55%)	11 (50%)	
Grade 2	22 (49%)	10 (45%)	11 (50%)	

Supplementary Table S1. Baseline laboratory parameters of the cohort.
Legend: Data were presented as mean ± standard deviation (SD) or as number (percentage) where indicated. ALT: alanine aminotransferase, AST: aspartate aminotransferase, GGT: gamma-glutamyl transferase, Hb: hemoglobin, WBC: leukocyte count, MPV: mean platelet volume, INR: International Normalized Ratio, PTT: prothrombin time, ALBI: Albumin-bilirubin. Albumin, GGT, and ALBI score were not available for 2 patients in the entire cohort. ALT, AST, INR, and PTT were not available for 1 patient in the entire cohort. P values were calculated using the Mann–Whitney U test. Statistically significant differences were indicated by asterisks (*p < 0.05, **p < 0.01, ***p < 0.001).

	CR	PR	SD	PD
Entire Cohort, n (%)	0	13 (29%)	19 (42%)	13 (29%)
iBT, n (%)	0	8 (35%)	7 (30%)	8 (35%)
cTACE/iBT, n (%)	0	5 (23%)	12 (55%)	5 (23%)

Supplementary Table S2. Target tumor response assessment at 8-week follow-up according to RECIST 1.1.
Legend: RECIST 1.1: Response Evaluation Criteria in Solid Tumors version 1.1; CR, complete response; PR, partial response; SD, stable disease; PD, progressive disease. Responders were defined as patients achieving CR or PR according to RECIST 1.1. Data not available for 1 patient in the entire cohort.

	CR	PR	SD	PD
Entire Cohort, n (%)	29 (67%)	3 (7%)	3 (7%)	8 (19%)
iBT, n (%)	15 (68%)	2 (9%)	1 (5%)	4 (18%)
cTACE/iBT, n (%)	14 (67%)	1 (5%)	2 (10%)	4 (19%)

Supplementary Table S3. Target tumor response assessment at 8-week follow-up according to mRECIST.
Legend: mRECIST: modified Response Evaluation Criteria in Solid Tumors; CR, complete response; PR, partial response; SD, stable disease; PD, progressive disease. Responders were defined as patients achieving CR or PR according to mRECIST. Data not available for 3 patients in the entire cohort.

	Nonviable	Equivocal	Viable
Entire Cohort, n (%)	29 (67%)	6 (14%)	8 (19%)
iBT, n (%)	15 (68%)	3 (14%)	4 (18%)
cTACE/iBT, n (%)	14 (67%)	3 (14%)	4 (19%)

Supplementary Table S4. Target tumor response assessment at 8-week follow-up according to LI-RADS TRA v2018.
Legend: LI-RADS TRA v2018: Liver Imaging and Data Reporting System Treatment Response Algorithm version 2018. Responders were defined as lesions classified as nonviable according to LI-RADS TRA. Data not available for 3 patients in the entire cohort.

	Entire Cohort (n = 46)	iBT (n = 23)	cTACE/iBT (n = 23)	P value
IFN-γ (pg/mL), mean, SEM	14.5 ± 2.78	14.3 ± 3.73	14.7 ± 4.22	0.7773
IL-10 (pg/mL), mean, SEM	0.704 ± 0.15	0.532 ± 0.07	0.877 ± 0.29	0.9265
IL-13 (pg/mL), mean, SEM	1.14 ± 0.19	1.21 ± 0.29	1.08 ± 0.24	0.9957
IL-1β (pg/mL), mean, SEM	0.241 ± 0.12	0.407 ± 0.24	0.083 ± 0.03	0.5841
IL-2 (pg/mL), mean, SEM	0.765 ± 0.08	0.659 ± 0.07	0.871 ± 0.13	0.2537
IL-4 (pg/mL), mean, SEM	0.029 ± 0.01	0.036 ± 0.01	0.023 ± 0.01	0.0364*
IL-6 (pg/mL), mean, SEM	4.20 ± 0.78	5.67 ± 1.42	2.73 ± 0.53	0.0322*
IL-8 (pg/mL), mean, SEM	52.9 ± 15.8	65.0 ± 30.0	40.7 ± 10.7	0.4960
TNF-α (pg/mL), mean, SEM	2.24 ± 0.20	2.21 ± 0.18	2.27 ± 0.36	0.4588
IL-17 (pg/mL), mean, SEM	3.95 ± 1.06	4.25 ± 1.87	3.65 ± 1.06	0.2822
IL-5 (pg/mL), mean, SEM	0.809 ± 0.06	0.842 ± 0.09	0.776 ± 0.09	0.4721
MCP-1 (pg/mL), mean, SEM	302.5 ± 18.6	298.4 ± 28.0	306.6 ± 25.2	0.6953
MIP-1α (pg/mL), mean, SEM	30.9 ± 7.88	28.1 ± 9.19	33.8 ± 13.0	0.4105
VEGF (pg/mL), mean, SEM	542.0 ± 63.9	575.3 ± 99.8	508.7 ± 81.4	0.6633
bFGF (pg/mL), mean, SEM	22.5 ± 6.31	18.7 ± 4.32	26.3 ± 12.0	0.9134

Supplementary Table S5. Baseline levels of cytokines in peripheral blood.
Legend: Data were presented as mean ± standard error of the mean (SEM). IFN-γ: interferon gamma; IL: interleukin; TNF-α: tumor necrosis factor alpha; MCP-1: monocyte chemoattractant protein-1; VEGF-A: vascular endothelial growth factor A; bFGF: basic fibroblast growth factor. Statistical comparisons between the iBT and cTACE/iBT groups were performed using the Mann–Whitney U test. Statistically significant differences were indicated by asterisks (*p < 0.05, **p < 0.01, ***p < 0.001).

	1-day prior cTACE	1-day post cTACE	p
IFN-γ (pg/mL)	14.68 ± 4.22	3.01 ± 1.22	****< 0.0001
IL-1β (pg/mL)	0.083 ± 0.03	0.107 ± 0.04	0.3333
IL-2 (pg/mL)	0.871 ± 0.13	0.862 ± 0.15	0.5202
IL-4 (pg/mL)	0.776 ± 0.09	0.431 ± 0.18	***0.0006
IL-5 (pg/mL)	0.023 ± 0.007	0.010 ± 0.003	**0.0089
IL-6 (pg/mL)	2.73 ± 0.53	8.08 ± 2.75	**0.0043
IL-8 (pg/mL)	40.75 ± 10.75	18.22 ± 2.78	**0.0049
IL-10 (pg/mL)	0.877 ± 0.29	0.848 ± 0.31	0.5399
IL-13 (pg/mL)	1.077 ± 0.24	0.855 ± 0.20	0.1089
IL-17 (pg/mL)	3.65 ± 1.06	2.21 ± 0.71	**0.0049
TNF-α (pg/mL)	2.27 ± 0.36	1.44 ± 0.13	***0.0003
MCP-1 (pg/mL)	306.6 ± 25.15	179.4 ± 19.9	****< 0.0001
MIP-1α (pg/mL)	33.75 ± 12.98	14.36 ± 1.36	***0.0003
VEGF (pg/mL)	508.7 ± 81.35	522.5 ± 75.74	0.9406
bFGF (pg/mL)	26.30 ± 11.95	25.69 ± 5.13	0.3564

Supplementary Table S6. Serum levels of cytokines 1 day prior to and 1 day after cTACE.
Legend: Data were presented as mean ± SEM. Statistical comparisons were performed using the Wilcoxon matched pairs signed-rank test. Statistically significant differences were indicated by asterisks (*p<0.05, **p<0.01, ***p<0.001).

	Untreated (pre)treated	1-day post cTACE	p
IFN-γ (pg/mL)	133.1 ± 23.03	133.5 ± 19.27	0.7607
IL-1β (pg/mL)	78.55 ± 34.79	15.67 ± 4.94	0.3181
IL-2 (pg/mL)	104.9 ± 15.33	73.77 ± 7.97	0.0952
IL-4 (pg/mL)	5.14 ± 1.07	6.07 ± 1.05	0.5028
IL-5 (pg/mL)	16.55 ± 4.20	10.66 ± 1.13	0.5313
IL-6 (pg/mL)	78.52 ± 10.24	60.59 ± 12.72	0.1044
IL-8 (pg/mL)	2305 ± 1531	1555 ± 953.3	0.6476
IL-10 (pg/mL)	30.76 ± 6.21	22.11 ± 2.50	0.6166
IL-13 (pg/mL)	368.8 ± 45.3	299.6 ± 40.3	0.2047
IL-17 (pg/mL)	30.94 ± 14.22	9.77 ± 2.62	*0.0381
TNF-α (pg/mL)	39.39 ± 7.67	21.06 ± 2.61	0.0614
MCP-1 (pg/mL)	280.8 ± 89.91	282.1 ± 120.2	0.4588
MIP-1α (pg/mL)	175.5 ± 28.86	138.6 ± 24.01	0.3567
VEGF (pg/mL)	1618 ± 1243	332.7 ± 167.3	0.0877
bFGF (pg/mL)	5843 ± 2321	8513 ± 3629	0.5276

Supplementary Table S7. TIF cytokine levels in untreated (iBT cohort) versus 1 day post-cTACE (cTACE/iBT cohort).
Legend: Data were presented as mean ± SEM. Statistical comparisons were performed using the Mann-Whitney U test. Statistically significant differences were indicated by asterisks (*p < 0.05, **p < 0.01, ***p < 0.001).

	Responder (n = 5)	Non-responder (n = 17)	p
IFN-γ (pg/mL)	11.61 ± 2.5	15.37 ± 5.69	0.4929
IL-1β (pg/mL)	0.13 ± 0.05	0.07 ± 0.04	0.5302
IL-2 (pg/mL)	0.73 ± 0.18	0.94 ± 0.17	0.7043
IL-4 (pg/mL)	0.01 ± 0.004	0.03 ± 0.01	0.1052
IL-5 (pg/mL)	0.66 ± 0.26	0.82 ± 0.1	0.3193
IL-6 (pg/mL)	2.46 ± 0.89	2.27 ± 0.38	0.9396
IL-8 (pg/mL)	29.79 ± 13.14	45.18 ± 14.04	0.6591
IL-10 (pg/mL)	1.46 ± 1.01	0.72 ± 0.28	0.4008
IL-13 (pg/mL)	0.95 ± 0.44	0.97 ± 0.26	0.7660
IL-17 (pg/mL)	3.32 ± 2.1	3.23 ± 1.21	0.5946
TNF-α (pg/mL)	2.15 ± 0.28	2.29 ± 0.48	0.8201
MCP-1 (pg/mL)	331.38 ± 53.2	307.23 ± 29.84	0.6486
MIP-1α (pg/mL)	22.05 ± 3.66	38.18 ± 17.55	0.8795
VEGF (pg/mL)	478.68 ± 149.3	530.02 ± 102.28	0.9396
bFGF (pg/mL)	11.58 ± 2.73	32.05 ± 16.03	0.7043

(a) Pre-treatment serum cytokine levels (cTACE/iBT cohort).

	Responder (n = 5)	Non-responder (n = 17)	p
IFN-γ (pg/mL)	1.18 ± 0.36	3.66 ± 1.63	0.7212
IL-1β (pg/mL)	0.08 ± 0.03	0.12 ± 0.05	0.6889
IL-2 (pg/mL)	0.64 ± 0.27	0.96 ± 0.19	0.1196
IL-4 (pg/mL)	0.002 ± 0.0006	0.012 ± 0.004	0.1654
IL-5 (pg/mL)	0.2 ± 0.11	0.52 ± 0.24	0.2546
IL-6 (pg/mL)	7.24 ± 3.14	8.66 ± 3.63	0.9396
IL-8 (pg/mL)	14.14 ± 3.2	19.85 ± 3.59	0.5946
IL-10 (pg/mL)	0.35 ± 0.03	0.99 ± 0.41	0.3193
IL-13 (pg/mL)	0.89 ± 0.34	0.81 ± 0.19	0.5517
IL-17 (pg/mL)	1.16 ± 0.39	2.28 ± 0.92	0.7616
TNF-α (pg/mL)	1.43 ± 0.36	1.41 ± 0.15	0.9396
MCP-1 (pg/mL)	214.48 ± 49.76	173.31 ± 22.6	0.4008
MIP-1α (pg/mL)	14.55 ± 2.83	13.88 ± 1.64	0.4837
VEGF (pg/mL)	543.78 ± 152.05	532.29 ± 93.19	0.8316
bFGF (pg/mL)	22.84 ± 19.28	25.84 ± 4.58	0.0849

(b) Post-treatment serum cytokine levels (cTACE/iBT cohort).

	Responder (n = 5)	Non-responder (n = 17)	p
IFN-γ (pg/mL)	137.23 ± 31.56	133.67 ± 24.81	0.7043
IL-1β (pg/mL)	11.6 ± 4.87	17.48 ± 6.53	0.8795
IL-2 (pg/mL)	74.28 ± 15.66	75.27 ± 9.85	0.9396
IL-4 (pg/mL)	10.46 ± 1.73	10.81 ± 1.47	0.8795
IL-5 (pg/mL)	8.08 ± 1.69	5.15 ± 1.26	0.1847
IL-6 (pg/mL)	54.62 ± 7.05	63.15 ± 17.2	0.6486
IL-8 (pg/mL)	978.81 ± 911.27	1805.85 ± 1269.76	0.8201
IL-10 (pg/mL)	21.24 ± 3.46	22.6 ± 3.27	0.9396
IL-13 (pg/mL)	276.71 ± 33.81	307.97 ± 53.97	0.8201
IL-17 (pg/mL)	8.92 ± 5.31	8.73 ± 2.97	0.9396
TNF-α (pg/mL)	24.57 ± 6.91	20.87 ± 2.85	0.5946
MCP-1 (pg/mL)	99.5 ± 45.29	346.42 ± 160.39	> 0.9999
MIP-1α (pg/mL)	187.12 ± 58.83	130.35 ± 27.05	0.3587
VEGF (pg/mL)	477.54 ± 402.96	306.16 ± 198.63	0.5021
bFGF (pg/mL)	2422.42 ± 1373.84	10462.42 ± 4839.17	0.7043

(c) TIF cytokine levels from treated tumors (cTACE/iBT cohort).

	Responder (n = 8)	Non-responder (n = 15)	p
IFN-γ (pg/mL)	9.84 ± 1.05	16.65 ± 5.67	0.8746
IL-1β (pg/mL)	0.46 ± 0.41	0.35 ± 0.29	0.2826
IL-2 (pg/mL)	0.64 ± 0.14	0.67 ± 0.09	0.7763
IL-4 (pg/mL)	0.04 ± 0.01	0.03 ± 0.01	0.1649
IL-5 (pg/mL)	0.84 ± 0.13	0.84 ± 0.12	0.9246
IL-6 (pg/mL)	7.79 ± 3.61	4.54 ± 1.07	0.6355
IL-8 (pg/mL)	28.88 ± 9.21	84.25 ± 45.42	0.1901
IL-10 (pg/mL)	0.69 ± 0.16	0.45 ± 0.06	0.3246
IL-13 (pg/mL)	0.69 ± 0.26	1.49 ± 0.41	0.2520
IL-17 (pg/mL)	1.66 ± 0.4	5.63 ± 2.82	0.8718
TNF-α (pg/mL)	2.58 ± 0.44	2.01 ± 0.15	0.1901
MCP-1 (pg/mL)	252.84 ± 31.63	322.77 ± 38.65	0.4281
MIP-1α (pg/mL)	18.53 ± 2.81	33.14 ± 14.01	0.4578
VEGF (pg/mL)	515.73 ± 107.08	607.04 ± 143.97	0.9748
bFGF (pg/mL)	7.59 ± 1.46	24.69 ± 6.1	0.0755

(d) Pre-treatment serum cytokine levels (iBT cohort).

	Responder (n = 8)	Non-responder (n = 15)	p
IFN-γ (pg/mL)	118.36 ± 46.6	147.58 ± 22.8	0.2667
IL-1β (pg/mL)	123.95 ± 79.66	13.43 ± 5.21	0.0704
IL-2 (pg/mL)	129.38 ± 37.83	96.4 ± 8.75	0.7639
IL-4 (pg/mL)	13.14 ± 3.57	13.47 ± 1.45	0.365
IL-5 (pg/mL)	5.35 ± 1.9	7.31 ± 1.68	0.7135
IL-6 (pg/mL)	88.15 ± 20.43	59.52 ± 5.64	0.5699
IL-8 (pg/mL)	1301.82 ± 686.83	141.72 ± 63.84	*0.0103
IL-10 (pg/mL)	29.23 ± 9.22	26.86 ± 2.31	0.2973
IL-13 (pg/mL)	373.0 ± 64.4	312.85 ± 33.87	0.5252
IL-17 (pg/mL)	13.87 ± 5.7	17.56 ± 4.42	0.8154
TNF-α (pg/mL)	39.78 ± 11.91	36.2 ± 5.64	0.9203
MCP-1 (pg/mL)	375.32 ± 114.54	281.44 ± 192.05	0.024
MIP-1α (pg/mL)	206.98 ± 51.43	145.13 ± 34.75	0.2382
VEGF (pg/mL)	818.56 ± 606.01	98.95 ± 43.59	*0.0185
bFGF (pg/mL)	3511.52 ± 1185.79	1830.96 ± 1247.9	0.0817

(e) TIF cytokine levels from untreated tumors (iBT cohort).

Supplementary Table S8. Association between RECIST response and cytokine levels in cTACE/iBT and iBT groups.

Legend: Data were presented as mean ± SEM. Comparisons between responders (CR + PR) and non-responders (SD + PD) were made using the Mann-Whitney U test. Statistically significant differences were indicated by asterisks (*p < 0.05, **p < 0.01, ***p < 0.001). Subtables show: (a) Pre-treatment serum cytokine levels (cTACE/iBT cohort), (b) Post-treatment serum cytokine levels (cTACE/iBT cohort), (c) TIF cytokine levels from treated tumors (cTACE/iBT cohort), (d) Pre-treatment serum cytokine levels (iBT cohort) and (e) TIF cytokine levels from untreated tumors (iBT cohort).

	**Responder (n = 15)**	**Non-responder (n = 6)**	**p**
IFN-γ (pg/mL)	10.65 ± 2.03	21.82 ± 15.67	0.6222
IL-1β (pg/mL)	0.07 ± 0.02	0.14 ± 0.11	0.8629
IL-2 (pg/mL)	0.88 ± 0.17	0.93 ± 0.26	0.5693
IL-4 (pg/mL)	0.02 ± 0.01	0.02 ± 0.005	0.9240
IL-5 (pg/mL)	0.77 ± 0.13	0.85 ± 0.13	0.4702
IL-6 (pg/mL)	2.58 ± 0.48	1.69 ± 0.33	0.4243
IL-8 (pg/mL)	30.37 ± 6.94	58.0 ± 36.15	0.5067
IL-10 (pg/mL)	0.81 ± 0.34	1.15 ± 0.77	0.9699
IL-13 (pg/mL)	0.73 ± 0.23	1.38 ± 0.53	0.3535
IL-17 (pg/mL)	3.88 ± 1.48	1.39 ± 0.16	0.2661
TNF-α (pg/mL)	1.87 ± 0.22	3.17 ± 1.25	0.2672
MCP-1 (pg/mL)	333.79 ± 33.67	249.6 ± 30.8	0.1537
MIP-1α (pg/mL)	20.37 ± 1.88	70.35 ± 49.52	0.4702
VEGF (pg/mL)	450.63 ± 70.58	485.98 ± 154.53	0.7910
bFGF (pg/mL)	33.74 ± 18.11	14.78 ± 5.22	0.7910

(a) Pre-treatment serum cytokine levels (cTACE/iBT cohort).

	**Responder (n = 15)**	**Non-responder (n = 6)**	**p**
IFN-γ (pg/mL)	3.25 ± 1.78	2.61 ± 1.67	0.2512
IL-1β (pg/mL)	0.08 ± 0.03	0.21 ± 0.12	0.3113
IL-2 (pg/mL)	0.93 ± 0.21	0.73 ± 0.21	0.9096
IL-4 (pg/mL)	0.006 ± 0.002	0.01 ± 0.007	0.8217
IL-5 (pg/mL)	0.46 ± 0.27	0.41 ± 0.14	0.1729
IL-6 (pg/mL)	6.55 ± 1.64	13.83 ± 9.92	> 0.9999
IL-8 (pg/mL)	16.7 ± 3.42	19.26 ± 5.07	0.5693
IL-10 (pg/mL)	0.56 ± 0.12	1.64 ± 1.16	0.9699
IL-13 (pg/mL)	0.79 ± 0.21	0.8 ± 0.28	0.723
IL-17 (pg/mL)	2.17 ± 0.96	0.73 ± 0.23	0.1781
TNF-α (pg/mL)	1.39 ± 0.16	1.4 ± 0.33	0.8500
MCP-1 (pg/mL)	195.84 ± 27.62	143.72 ± 26.96	0.2672
MIP-1α (pg/mL)	12.82 ± 1.48	14.42 ± 2.29	0.1991
VEGF (pg/mL)	478.56 ± 80.69	545.74 ± 163.38	0.8960
bFGF (pg/mL)	19.43 ± 6.59	38.18 ± 9.15	*0.0449

(b) Post-treatment serum cytokine levels (cTACE/iBT cohort).

	Responder (n = 15)	Non-responder (n = 6)	p
IFN-γ (pg/mL)	143.72 ± 27.78	121.27 ± 25.42	0.9699
IL-1β (pg/mL)	16.32 ± 7.18	17.63 ± 6.4	0.4702
IL-2 (pg/mL)	79.82 ± 10.55	55.67 ± 10.55	0.2672
IL-4 (pg/mL)	10.99 ± 1.56	10.03 ± 2.1	0.7333
IL-5 (pg/mL)	5.4 ± 1.19	7.82 ± 2.33	0.4702
IL-6 (pg/mL)	69.62 ± 19.14	42.56 ± 6.88	0.1781
IL-8 (pg/mL)	1844.26 ± 1407.73	1316.03 ± 1122.57	0.6768
IL-10 (pg/mL)	23.76 ± 3.34	17.01 ± 4.15	0.3023
IL-13 (pg/mL)	323.07 ± 58.06	256.36 ± 53.95	0.8500
IL-17 (pg/mL)	8.29 ± 3.36	9.4 ± 4.38	0.7333
TNF-α (pg/mL)	22.09 ± 3.5	17.96 ± 2.99	0.4243
MCP-1 (pg/mL)	323.19 ± 177.42	256.26 ± 136.21	0.7333
MIP-1α (pg/mL)	135.18 ± 30.48	182.28 ± 45.7	0.3403
VEGF (pg/mL)	421.46 ± 250.21	209.9 ± 145.73	0.8456
bFGF (pg/mL)	8011.62 ± 3975.64	11616.86 ± 10337.33	0.9699

(c) TIF cytokine levels from treated tumors (cTACE/iBT cohort).

	Responder (n = 17)	Non-responder (n = 5)	p
IFN-γ (pg/mL)	17.41 ± 4.84	5.54 ± 1.65	*0.0193
IL-1β (pg/mL)	0.26 ± 0.19	0.91 ± 0.88	0.7028
IL-2 (pg/mL)	0.72 ± 0.09	0.5 ± 0.16	0.2177
IL-4 (pg/mL)	0.04 ± 0.01	0.01 ± 0.01	*0.0121
IL-5 (pg/mL)	0.9 ± 0.09	0.74 ± 0.26	0.4008
IL-6 (pg/mL)	6.92 ± 1.83	2.44 ± 0.73	0.0849
IL-8 (pg/mL)	72.37 ± 40.38	51.98 ± 15.96	0.2827
IL-10 (pg/mL)	0.61 ± 0.09	0.33 ± 0.08	0.1012
IL-13 (pg/mL)	0.96 ± 0.24	2.3 ± 0.96	0.1340
IL-17 (pg/mL)	2.19 ± 0.54	11.42 ± 8.24	0.4837
TNF-α (pg/mL)	2.4 ± 0.21	1.88 ± 0.28	0.1636
MCP-1 (pg/mL)	296.0 ± 33.55	323.46 ± 62.78	0.4929
MIP-1α (pg/mL)	32.45 ± 12.34	16.62 ± 0.7	0.3901
VEGF (pg/mL)	629.16 ± 126.56	484.27 ± 137.54	0.8201
bFGF (pg/mL)	11.07 ± 2.58	46.1 ± 11.96	*0.0114

(d) Pre-treatment serum cytokine levels (iBT cohort).

	Responder (n = 17)	Non-responder (n = 5)	p
IFN-γ (pg/mL)	152.2 ± 29.32	93.39 ± 15.76	0.3587
IL-1β (pg/mL)	55.61 ± 29.4	170.26 ± 126.8	0.4929
IL-2 (pg/mL)	110.6 ± 19.42	96.72 ± 24.98	0.7043
IL-4 (pg/mL)	13.8 ± 2.23	27.39 ± 18.46	0.8795
IL-5 (pg/mL)	7.27 ± 1.37	6.21 ± 1.74	0.7503
IL-6 (pg/mL)	81.88 ± 10.94	69.8 ± 31.48	0.4456
IL-8 (pg/mL)	2726 ± 2060	1327 ± 943.4	0.5426
IL-10 (pg/mL)	28.07 ± 4.97	43.67 ± 23.96	0.8795
IL-13 (pg/mL)	355.6 ± 45.52	431.19 ± 148.9	> 0.9999
IL-17 (pg/mL)	17.17 ± 4.84	80.1 ± 63.44	0.2177
TNF-α (pg/mL)	38.33 ± 7	48.71 ± 27.52	0.7043
MCP-1 (pg/mL)	338.8 ± 118.4	106.42 ± 51.1	0.5946
MIP-1α (pg/mL)	193.1 ± 35.83	112.12 ± 48.34	0.2124
VEGF (pg/mL)	2076 ± 1679	230.35 ± 159.9	0.9396
bFGF (pg/mL)	5948 ± 2983	5530 ± 3905	0.7616

(e) TIF cytokine levels from untreated tumors (iBT cohort).

Supplementary Table S9. Association between mRECIST response and cytokine levels in cTACE/iBT and iBT groups.

Legend: Data were presented as mean ± SEM. Comparisons between responders (CR + PR) and non-responders (SD + PD) were made using the Mann-Whitney U test. Statistically significant differences were indicated by asterisks (*p < 0.05, **p < 0.01, ***p < 0.001). Subtables show: (a) Pre-treatment serum cytokine levels (cTACE/iBT cohort), (b) Post-treatment serum cytokine levels (cTACE/iBT cohort), (c) TIF cytokine levels from treated tumors (cTACE/iBT cohort), (d) Pre-treatment serum cytokine levels (iBT cohort) and (e) TIF cytokine levels from untreated tumors (iBT cohort).

Baseline Levels of Blood Cytokines			
	**Homogeneous (n = 7)**	**Heterogeneous (n = 16)**	**p**
IFN-γ (pg/mL)	10.1 ± 2.04	16.7 ± 5.99	0.9221
IL-10 (pg/mL)	0.60 ± 0.14	0.997 ± 0.41	0.4515
IL-13 (pg/mL)	1.82 ± 0.53	0.75 ± 0.22	0.1063
IL-1β (pg/mL)	0.047 ± 0.027	0.10 ± 0.043	0.4599
IL-2 (pg/mL)	1.03 ± 0.36	0.8 ± 0.1	*0.0266
IL-4 (pg/mL)	0.038 ± 0.02	0.017 ± 0.005	0.1914
IL-6 (pg/mL)	4.05 ± 1.4	2.15 ± 0.404	0.1181
IL-8 (pg/mL)	25.4 ± 8.7	47.5 ± 14.9	0.3760
TNF-α (pg/mL)	2.2 ± 0.24	2.3 ± 0.51	0.5787
IL-17 (pg/mL)	3.7 ± 1.51	3.6 ± 0.40	0.4127
IL-5 (pg/mL)	0.80 ± 0.22	0.76 ± 0.09	0.7198
MCP-1 (pg/mL)	256.7 ± 43.9	328.4 ± 29.9	0.1353
MIP-1α (pg/mL)	18.6 ± 1.08	40.4 ± 18.6	0.6714
VEGF (pg/mL)	341 ± 88.8	582 ± 106.7	0.1977
bFGF (pg/mL)	11 ± 3.51	33 ± 17	0.3680
**Post-cTACE Blood Levels**			
	**Homogeneous (n = 7)**	**Heterogeneous (n = 16)**	**p**
IFN-γ (pg/mL)	2.41 ± 1.61	3.26 ± 3.27	0.9106
IL-10 (pg/mL)	0.62 ± 0.14	0.95 ± 0.44	0.4921
IL-13 (pg/mL)	1.66 ± 0.49	0.50 ± 0.12	0.0695
IL-1β (pg/mL)	0.02 ± 0.007	0.14 ± 0.05	*0.0312
IL-2 (pg/mL)	0.97 ± 0.45	0.82 ± 0.11	0.4127
IL-4 (pg/mL)	0.005 ± 0.002	0.012 ± 0.004	0.5450
IL-6 (pg/mL)	6.26 ± 1.95	8.87 ± 3.9	0.7692
IL-8 (pg/mL)	12.5 ± 3	20.7 ± 3.6	0.2490
TNF-α (pg/mL)	1.58 ± 0.27	1.38 ± 0.15	0.5346
IL-17 (pg/mL)	2.18 ± 0.76	2.22 ± 0.98	0.3084
IL-5 (pg/mL)	0.28 ± 0.10	0.497 ± 0.25	0.8309
MCP-1 (pg/mL)	150.7 ± 45.4	192 ± 20.96	0.1353
MIP-1α (pg/mL)	13.7 ± 2.7	14.7 ± 1.63	0.5249
VEGF (pg/mL)	313.5 ± 127.3	614 ± 86.2	*0.0318
bFGF (pg/mL)	18.1 ± 6.8	29 ± 6.7	0.5346
**TIF Levels of Cytokines**			
	**Homogeneous (n = 7)**	**Heterogeneous (n = 16)**	**p**
IFN-γ (pg/mL)	122.2 ± 16.7	138.4 ± 27	0.9221
IL-10 (pg/mL)	19.5 ± 2.2	23.3 ± 3.5	0.6244

IL-13 (pg/mL)	260.6 ± 35.7	316.7 ± 55.9	0.8195
IL-1β (pg/mL)	7.3 ± 2.03	19.3 ± 6.9	0.2167
IL-2 (pg/mL)	62.2 ± 8.6	78.8 ± 10.7	0.3760
IL-4 (pg/mL)	9.5 ± 1.2	11.2 ± 1.6	0.5025
IL-6 (pg/mL)	47.9 ± 6.9	66.1 ± 18.1	0.7804
IL-8 (pg/mL)	194 ± 100.5	2150 ± 1355	0.6714
TNF-α (pg/mL)	16.5 ± 3.8	23.2 ± 3.3	0.3163
IL-17 (pg/mL)	17.5 ± 6.8	6.4 ± 1.96	0.1581
IL-5 (pg/mL)	4.7 ± 1.63	6.7 ± 1.33	0.4580
MCP-1 (pg/mL)	175.8 ± 141.2	328.7 ± 162.7	0.4127
MIP-1α (pg/mL)	133.1 ± 50.2	141 ± 27.8	0.5787
VEGF (pg/mL)	86.7 ± 58.8	440 ± 236.4	0.5888
bFGF (pg/mL)	8661 ± 6580	8449 ± 4494	0.9221

Supplementary Table S10. Association between Lipiodol deposition patterns and cytokine levels in serum and TIF.

Legend: Data were presented as mean ± SEM. Comparisons between homogeneous and heterogeneous Lipiodol deposition groups were performed using Mann–Whitney U test. Statistically significant differences were indicated by asterisks (*p < 0.05, **p < 0.01, ***p < 0.001). Subtables show: Baseline serum cytokine levels before cTACE, post-treatment serum cytokine levels (1-day post-cTACE) and TIF cytokine levels from treated tumors.

	**Tumor only**	**1 segment**	**> 2 segments**
1 day after cTACE (n, %)	6 (26%)	14 (61%)	3 (13%)

(a) Frequency (%) of Lipiodol distribution patterns (tumor-only, single segment, > 2 segments) assessed 1 day after cTACE.

**Baseline Levels of Blood Cytokines**			
	**Tumoral (n = 6)**	**Segmental (n = 17)**	**p**
IFN-γ (pg/mL)	28.3 ± 15	9.88 ± 1.7	0.3543
IL-10 (pg/mL)	1.47 ± 0.72	0.76 ± 0.3	*0.0243
IL-13 (pg/mL)	1.07 ± 0.39	1.14 ± 0.3	> 0.9999
IL-1β (pg/mL)	0.029 ± 0.001	0.102 ± 0.04	0.5517
IL-2 (pg/mL)	1.45 ± 0.4	0.67 ± 0.07	0.1351
IL-4 (pg/mL)	0.04 ± 0.02	0.02 ± 0.005	0.1396
IL-6 (pg/mL)	3.75 ± 0.73	2.37 ± 0.65	**0.0033
IL-8 (pg/mL)	40.8 ± 12.2	40.7 ± 14.1	0.5162
TNF-α (pg/mL)	2.42 ± 0.35	2.22 ± 0.47	0.1351
IL-17 (pg/mL)	5.3 ± 3.37	3.1 ± 0.87	0.4727
IL-5 (pg/mL)	0.93 ± 0.24	0.72 ± 0.09	0.8115
MCP-1 (pg/mL)	302.5 ± 66.8	308 ± 26.2	0.6576
MIP-1α (pg/mL)	23.3 ± 3.38	37.5 ± 17.6	0.4727
VEGF (pg/mL)	469 ± 138	523 ± 101	0.9729
bFGF (pg/mL)	13.5 ± 4.39	30.8 ± 16.1	0.9616

Post-cTACE Blood Levels			
	**Tumoral Deposition (n = 6)**	**Segmental Deposition (n = 17)**	**p**
IFN-γ (pg/mL)	8.04 ± 4.1	1.23 ± 0.42	0.1795
IL-10 (pg/mL)	2.1 ± 1.07	0.41 ± 0.07	***0.0006
IL-13 (pg/mL)	1.08 ± 0.42	0.78 ± 0.23	0.5459
IL-1β (pg/mL)	0.12 ± 0.06	0.10 ± 0.05	0.7213
IL-2 (pg/mL)	1.44 ± 0.47	0.66 ± 0.1	0.0736
IL-4 (pg/mL)	0.016 ± 0.003	0.008 ± 0.004	**0.0018
IL-6 (pg/mL)	8.85 ± 3.03	7.81 ± 3.6	0.3543
IL-8 (pg/mL)	23.3 ± 6.59	16.5 ± 2.96	0.2557
TNF-α (pg/mL)	1.93 ± 0.28	1.27 ± 0.13	*0.0197
IL-17 (pg/mL)	3.87 ± 2.31	1.62 ± 0.51	0.1770
IL-5 (pg/mL)	0.97 ± 0.64	0.24 ± 0.06	*0.0375
MCP-1 (pg/mL)	183 ± 58.1	178 ± 19.1	0.5162
MIP-1α (pg/mL)	15.4 ± 2.9	14 ± 1.6	0.6973
VEGF (pg/mL)	487 ± 156	535 ± 89	0.7702
bFGF (pg/mL)	14.3 ± 3.8	29.7 ± 6.6	0.3543
**TIF Levels of Cytokines**			
	**Tumoral Deposition (n = 6)**	**Segmental Deposition (n = 17)**	**p**
IFN-γ (pg/mL)	112.5 ± 15.8	141 ± 25.5	0.6088
IL-10 (pg/mL)	23.4 ± 3.8	21.7 ± 3.2	0.5162
IL-13 (pg/mL)	329 ± 107	289 ± 42	0.6576
IL-1β (pg/mL)	31.4 ± 17.2	10.1 ± 2.3	0.2927
IL-2 (pg/mL)	83.1 ± 14.2	70.5 ± 9.7	0.5617
IL-4 (pg/mL)	10.7 ± 1.9	10.7 ± 1.4	0.9051
IL-6 (pg/mL)	95.6 ± 46.2	48.2 ± 5.4	0.2799
IL-8 (pg/mL)	3722 ± 3476	790 ± 466	0.3917
TNF-α (pg/mL)	22.7 ± 6	20.6 ± 2.9	0.7719
IL-17 (pg/mL)	5.1 ± 2.7	11.4 ± 3.4	0.5518
IL-5 (pg/mL)	5.3 ± 2.8	6.4 ± 4.4	0.5048
MCP-1 (pg/mL)	709 ± 411	132 ± 52.9	0.1351
MIP-1α (pg/mL)	124 ± 59.3	144 ± 26	0.6088
VEGF (pg/mL)	646 ± 545	222 ± 128	0.2927
bFGF (pg/mL)	14948 ± 9231	6242 ± 3724	> 0.9999

(b) Tumoral vs Segmental Distribution (one segment and more than two segments included). Data were presented as mean ± SEM. Mann-Whitney U test was used.

Supplementary Table S11. Association between Lipiodol distribution patterns and cytokine levels in serum and TIF.

Legend: (a) Frequency (%) of Lipiodol distribution patterns assessed 1 day after conventional transarterial chemoembolization (cTACE), categorized as tumor-only deposition, segmental deposition

(one or more segments), or deposition involving more than two segments. (b) Comparison of baseline serum cytokine levels, post-cTACE serum cytokine levels, and TIF cytokine levels between patients with tumoral Lipiodol deposition and those with segmental Lipiodol deposition (including one segment and more than two segments). Data are presented as mean ± standard error of the mean (SEM). Comparisons between groups were performed using the Mann–Whitney U test. Statistically significant differences were indicated by asterisks (*p < 0.05, **p < 0.01, ***p < 0.001).

	OS	
**Univariate Analysis** / Variables	Hazard ratio (95% CI)	p value
**Patients Characteristics**		
Male (v. female)	2.14 (0.614 – 7.47)	0.2323
Age (≤ 70 vs > 70 yr)	1.05 (0.996 – 1.12)	*0.0682
**Tumor Characteristics**		
BCLC (A vs B)	1.77 (0.700 – 4.47)	0.2277
Number of viable tumor (Single vs Multiple)	0.56 (0.201 – 1.56)	0.2666
Long axis of target tumor (< 30 vs ≥ 30 mm)	2.94 (1.161 – 7.44)	*0.0229
**Baseline Laboratory Features**		
Total bilirubin (≤ 0.8 vs > 0.8 mg/dL)	1.498 (0.598 – 3.75)	0.3880
Albumin (≤ 39.55 vs > 39.55 g/L)	0.65 (0.262 – 1.63)	0.3626
ALT (≤ 32 vs > 32 U/L)	2.11 (0.814 – 5.47)	0.1246
AST (≤ 39 vs > 39 U/L)	3.904 (1.351 – 11.284)	*0.0119
INR (≤1.03 vs > 1.03)	2.32 (0.912 – 5.9)	*0.0774
ALBI Grade (1 vs 2)	1.51 (0.611 - 3.71)	0.3730
ALBI Score (< -2.67 vs ≥ -2.67)	2.07 (0.813 – 5.29)	0.1270
**Baseline Levels of Blood Cytokines**		
IFN-γ (high vs low median = 9.58 pg/mL)	2.63 (0.996 - 6.92)	*0.0508
IL-1β (high vs low median = 0.023 pg/mL)	2.36 (0.921 – 6.04)	*0.0738
IL-2 (high vs low median = 0.62 pg/mL)	1.46 (0.584 - 3.64)	0.4195

IL-6<br>(high vs low median = 2.35 pg/mL)	2.76<br>(0.991 – 7.68)	*0.0521
IL-8<br>(high vs low median = 24.7 pg/mL)	2.86<br>(1.073 – 7.61)	*0.0357
TNF-ɑ<br>(high vs low median = 1.98 pg/mL)	1.546<br>(0.621 – 3.85)	0.3494
MCP-1<br>(high vs low median = 275 pg/mL)	2.13<br>(0.823 – 5.504)	0.1193
MIP-1ɑ<br>(high vs low median = 17.8 pg/mL)	1.54<br>(0.616 – 3.827)	0.3570
IL-4<br>(high vs low median = 0.017 pg/mL)	1.06<br>(0.429 – 2.62)	0.8988
IL-5<br>(high vs low median = 0.68 pg/mL)	0.939<br>(0.381 – 2.32)	0.8918
IL-10<br>(high vs low median = 0.466 pg/mL)	0.740<br>(0.298 – 1.84)	0.5174
IL-13<br>(high vs low median = 0.88 pg/mL)	1.77<br>(0.695 – 4.50)	0.232
IL-17<br>(high vs low median = 1.63 pg/mL)	2.57<br>(1.005 – 6.56)	*0.0488
VEGF<br>(high vs low median = 411 pg/mL)	2.48<br>(0.937 – 6.547)	*0.0673
bFGF<br>(high vs low median = 10.4 pg/mL)	1.54<br>(0.616 – 3.827)	0.3579
**ROI Analysis**		
Arterial Phase Ratio	0.617<br>(0.247 – 1.54)	0.3017
Portal Phase Ratio	0.929<br>(0.238 – 1.48)	0.2623
Venous Phase Ratio	0.352<br>(0.134 – 0.929)	*0.0350
DWI Ratio	0.716<br>(0.287 – 1.78)	0.4726
ADC Ratio	1.55<br>(0.624 – 3.87)	0.3446
T2 Ratio	0.864<br>(0.344 – 2.17)	0.7549
HBP Ratio	1.05<br>(0.426 - 2.59)	0.9132

Supplementary Table S12. Univariate analysis of overall survival (OS) in the entire cohort (n = 46).

Legend: Hazard ratios (HR) with 95% confidence intervals (CI) were estimated using Cox proportional hazards regression. Survival distributions were compared using the log-rank test. Variables include patient characteristics, tumor characteristics, baseline laboratory features, baseline serum cytokine levels, and MRI ROI-based signal intensity ratios. Cytokine levels were dichotomized into high vs. low groups based on median values. Significant results (p < 0.1) were indicated with an asterisk (*).

Model 1							
**Variable**	**Coefficient**	**Standard Error**	**HR**	**95% CI (Lower)**	**95% CI (Upper)**	**z**	**p**
AST	0.823	0.602	2.28	0.700	7.411	1.368	0.1713
Long axis	0.969	0.562	2.63	0.876	7.933	1.725	0.0846
IL-8	0.935	0.563	2.55	0.844	7.678	1.659	0.0970
IL-17	0.595	0.535	1.81	0.635	5.174	1.112	0.2662
Venous Phase	-1.002	0.533	0.37	0.129	1.044	-1.880	0.0601
**Model 2**							
**Variable**	**Coefficient**	**Standard Error**	**HR**	**95% CI (Lower)**	**95% CI (Upper)**	**z**	**p**
AST	1.023	0.584	2.78	0.885	8.746	1.751	0.0799
IL-8	0.470	0.554	1.60	0.540	4.737	0.849	0.3961
IL-17	0.614	0.496	1.85	0.698	4.886	1.236	0.2163

Supplementary Table S13. Multivariate analysis of overall survival (OS) in the entire cohort (n = 46).
Legend: Multivariate Cox proportional hazards regression analyses were performed to identify independent predictors of OS in the entire cohort. Variables were selected based on univariate screening, clinical relevance, and imaging parameters, taking into account potential collinearity and the limited number of events. Two multivariate Cox proportional hazards regression models were constructed to evaluate independent predictors of OS. Statistical significance in multivariable Cox regression analyses was defined as $p<0.1$. Model 1 included AST, target tumor long-axis diameter, IL-8, IL-17, and venous phase ROI ratio. Model 2 included AST, IL-8, and IL-17. Hazard ratios (HR) with 95% confidence intervals (CI) were reported.

	OS	
**Univariate Analysis**<br>Variables	Hazard ratio (95% CI)	p value
**Patients Characteristics**		
Male (v. female)	1.876 (0.48 – 7.33)	0.3653
Age (≤ 70 vs > 70 yr)	1.083 (0.01 – 1.16)	*0.0297
**Tumor Characteristics**		
BCLC (A vs B)	1.689 (0.196 – 14.57)	0.6336
Number of viable tumor (Single vs Multiple)	0.576 (0.12 – 2.73)	0.4864
Target tumor size (< 30 vs ≥ 30 mm)	3.314 (0.79 – 13.89)	0.1013
**Baseline Laboratory Features**		
Total bilirubin (≤ 0.63 vs > 0.63 mg/dL)	1.017 (0.29 – 3.52)	0.9788
Albumin (≤ 40.25 vs > 40.25 g/L)	0.899 (0.26 – 3.11)	0.8665
ALT (≤ 26.5 vs > 26.5 U/L)	0.893 (0.26 – 3.1)	0.8586
AST (≤ 38 vs > 38 U/L)	3.03 (0.835 – 10.99)	*0.0918
INR (≤ 1.03 vs >1.03)	1.713 (0.482 – 6.09)	0.4053
ALBI Grade (1 vs 2)	0.856 (0.24 – 3.04)	0.8665
ALBI Score (< -2.73 vs ≥ -2.73)	1.112 (0.32 – 3.85)	0.8100
**Baseline Levels of Blood Cytokines**		
IFN-γ (high vs low median = 10.01 pg/mL)	1.792 (0.505 – 6.37)	0.3670
IL-1β (high vs low median = 0.018 pg/mL)	1.67 (0.468 – 5.96)	0.4295
IL-2 (high vs low median = 0.53 pg/mL)	0.965 (0.276 – 3.38)	0.9559

IL-6<br>(high vs low median = 3.19 pg/mL)	3.09<br>(0.793 – 12.04)	0.1041
IL-8<br>(high vs low median = 29.9 pg/mL)	4.998<br>(1.05 – 23.83)	*0.0435
TNF-α<br>(high vs low median = 1.76 pg/mL)	0.997<br>(0.288–3.46)	0.9962
MCP-1<br>(high vs low median = 279 pg/mL)	6.48<br>(1.302 – 32.25)	*0.0225
MIP-1α<br>(high vs low median = 18.8 pg/mL)	1.802<br>(0.505 – 6.43)	0.3543
IL-17<br>(high vs low median = 1.78 pg/mL)	1.412<br>(0.405 – 4.92)	0.5885
IL-5<br>(high vs low median = 0.64 pg/mL)	1.332<br>(0.374 – 4.75)	0.6578
IL-4<br>(high vs low median = 0.013 pg/mL)	0.407<br>(0.11 – 1.50)	0.1771
IL-10<br>(high vs low median = 0.47 pg/mL)	0.316<br>(0.081–1.23)	*0.0970
IL-13<br>(high vs low median = 1.15 pg/mL)	6.96<br>(1.456–33.3)	*0.0151
VEGF<br>(high vs low median = 367 pg/mL)	2.93<br>(0.741–11.58)	0.1255
bFGF<br>(high vs low median = 10.7 pg/mL)	2.73<br>(0.747–9.96)	0.1288
**TIF Levels of Cytokines**		
IFN-γ<br>(high vs low median = 114.6 pg/mL)	0.157<br>(0.031 – 0.781)	*0.0238
IL-1β<br>(high vs low median = 9.06 pg/mL)	4.67<br>(1.18 – 18.44)	*0.0279
IL-2<br>(high vs low median = 95.7 pg/mL)	0.552<br>(0.155 – 1.96)	0.3581
IL-6<br>(high vs low median = 72.3 pg/mL)	0.939<br>(0.268 – 3.29)	0.9220
IL-8<br>(high vs low median = 160.1 pg/mL)	10.74<br>(1.34 – 85.93)	*0.0252
TNF-α<br>(high vs low median = 24.83 pg/mL)	1.36<br>(0.379 – 4.85)	0.6403
MCP-1<br>(high vs low median = 103.2 pg/mL)	2.08<br>(0.581 – 7.45)	0.2601
MIP-1α<br>(high vs low median = 169.2 pg/mL)	1.696<br>(0.471 – 6.11)	0.4192
IL-17<br>(high vs low median = 13.5 pg/mL)	0.492<br>(0.137 – 1.76)	0.2761
IL-5<br>(high vs low median = 6.9 pg/mL)	0.184<br>(0.046 – 0.743)	*0.0174
IL-4<br>(high vs low median = 10.6 pg/mL)	0.330<br>(0.081 – 1.34)	0.1201

IL-10<br>(high vs low median = 21.04 pg/mL)	0.441<br>(0.12 – 1.62)	0.2175
IL-13<br>(high vs low median = 282.1 pg/mL)	1.17<br>(0.338 – 4.08)	0.8002
VEGF<br>(high vs low median = 71.17 pg/mL)	4.598<br>(0.975 – 21.69)	*0.0539
bFGF<br>(high vs low median = 1975 pg/mL)	4.18<br>(0.886 – 19.72)	*0.0709
**ROI Analysis**		
Arterial Phase Ratio	1.264<br>(0.356 – 4.492)	0.7174
Portal Phase Ratio	0.403<br>(0.104 – 1.562)	0.1886
Venous Phase Ratio	0.447<br>(0.115 – 1.732)	0.2437
DWI Ratio	1.137<br>(0.320 – 4.040)	0.8429
ADC Ratio	1.932<br>(0.541 – 6.903)	0.3105
T2 Ratio	0.602<br>(0.169 – 2.142)	0.4336
HBP Ratio	1.112<br>(0.321 - 3.854)	0.8665

Supplementary Table S14. Univariate analyses of overall survival (OS) in the iBT cohort (n = 23).

Legend: Hazard ratios (HR) with 95% confidence intervals (CI) were calculated using the log-rank test. Cytokine levels were dichotomized into high vs low groups based on the median value. Significant results (p < 0.1) were indicated with an asterisk (*).

Model 1							
**Variable**	**Coefficient**	**Standard Error**	**HR**	**95% CI (Lower)**	**95% CI (Upper)**	**z**	**p**
Age	0.047	0.070	1.05	0.914	1.201	0.672	0.5019
IL-13 (serum)	1.050	1.582	2.86	0.129	63.545	0.664	0.5069
IL-8 (serum)	2.004	1.591	7.42	0.328	167.809	1.260	0.2077
MCP-1 (serum)	1.017	1.351	2.76	0.196	39.032	0.753	0.4517
IFN-γ (TIF)	-0.505	2.027	0.60	0.011	32.072	-0.249	0.8031
IL-1β (TIF)	3.294	1.269	26.93	2.240	324	2.595	*0.0094
IL-8 (TIF)	-0.340	2.110	0.71	0.011	44.512	-0.161	0.8721
IL-5 (TIF)	-1.505	1.414	0.22	0.014	3.548	-1.064	0.2872
**Model 2**							
**Variable**	**Coefficient**	**Standard Error**	**HR**	**95% CI (Lower)**	**95% CI (Upper)**	**z**	**p**
Age	0.119	0.050	1.13	1.021	1.243	2.380	*0.0173
IL-13 (serum)	2.734	1.201	15.39	1.437	164.92	2.260	*0.0238
IL-8 (serum)	-0.649	1.183	0.52	0.051	5.304	-0.549	0.5829
**Model 3**							
**Variable**	**Coefficient**	**Standard Error**	**HR**	**95% CI (Lower)**	**95% CI (Upper)**	**z**	**p**
Age	0.112	0.049	1.12	1.016	1.231	2.294	*0.0218
IL-13 (serum)	3.077	1.082	21.70	2.599	181.2	2.842	*0.0045
IL-1β (TIF)	2.243	0.958	9.42	1.441	61.58	2.341	*0.0192

Supplementary Table S15. Multivariate Cox regression analysis of overall survival (OS) in the iBT cohort (n = 23).

Legend: Multivariate Cox proportional hazards regression analyses were performed to identify independent predictors of OS in the iBT cohort. Variables were selected based on univariate screening ($p < 0.1$), clinical relevance, and consideration of the limited number of events to minimize overfitting. Model 1 included a broader set of selected serum and TIF cytokines together with age, whereas Models 2 and 3 represent reduced models focusing on clinically and biologically relevant parameters. Results were presented as hazard ratios (HR) with 95% confidence intervals (CI). Statistical significance was defined as $p<0.05$ and indicated by an asterisk (*).

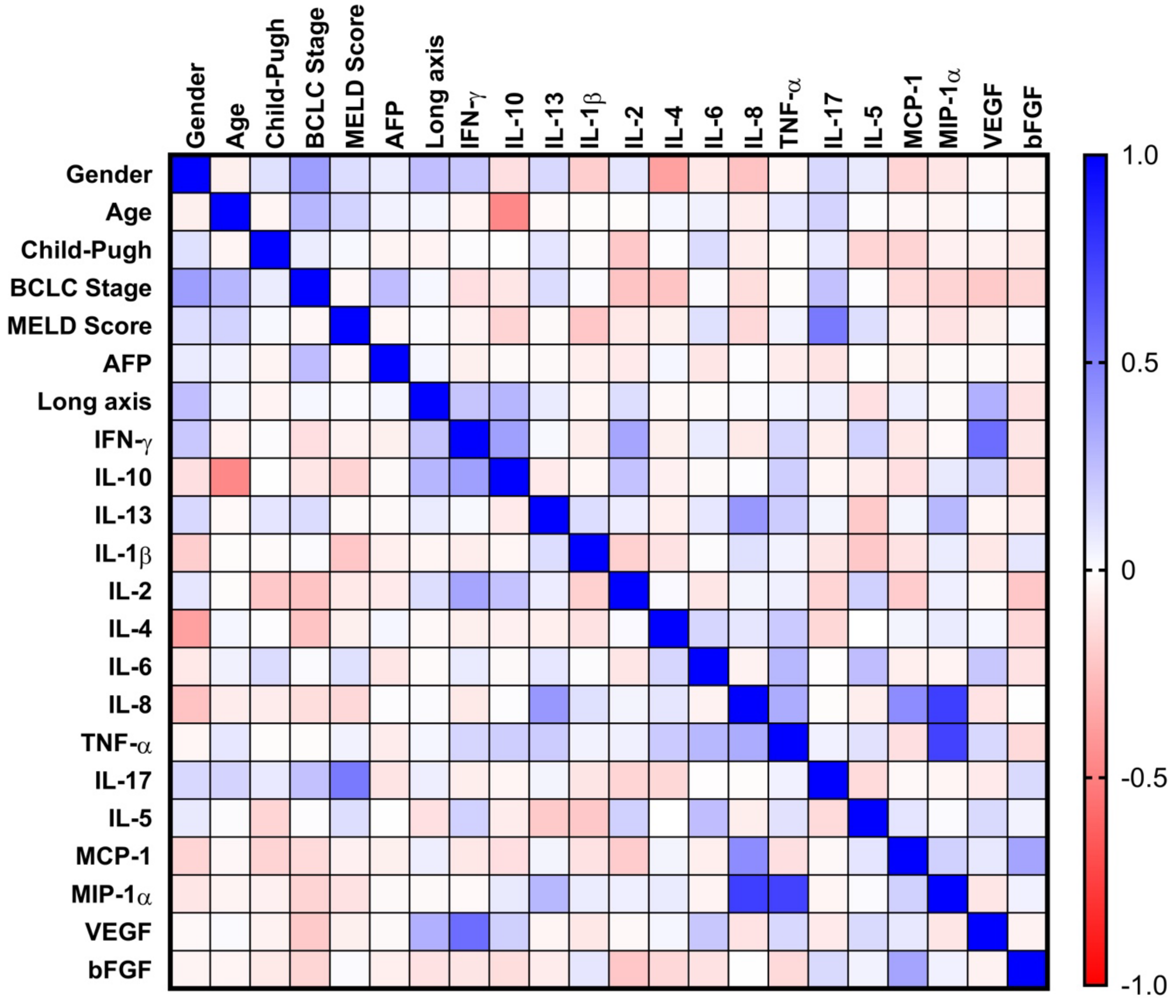

Supplementary Figure S1. Correlation matrix of baseline clinical characteristics and serum cytokine levels in the entire cohort.

Legend: Heatmap illustrating Pearson correlation coefficients between baseline patient characteristics, tumor-related variables, and baseline serum cytokine levels in the entire cohort (n = 46). Included variables comprise demographic parameters (age, gender), liver function and disease severity scores (Child–Pugh class, MELD score, BCLC stage), tumor-related features (AFP, target tumor long-axis diameter), and circulating cytokines measured at baseline. Color intensity represents the strength and direction of correlations, with blue indicating positive correlations and red indicating negative correlations. Pearson correlation coefficients range from −1 to +1, as shown by the color scale. All correlations were calculated using Pearson's correlation test. (AFP, alpha-fetoprotein; BCLC, Barcelona Clinic Liver Cancer; MELD, Model for End-Stage Liver Disease; IFN-γ, interferon gamma; IL, interleukin; TNF-α, tumor necrosis factor alpha; MCP-1, monocyte chemoattractant protein 1; MIP-1α, macrophage nflammatory protein 1 alpha; VEGF, vascular endothelial growth factor; bFGF, basic fibroblast growth factor.)

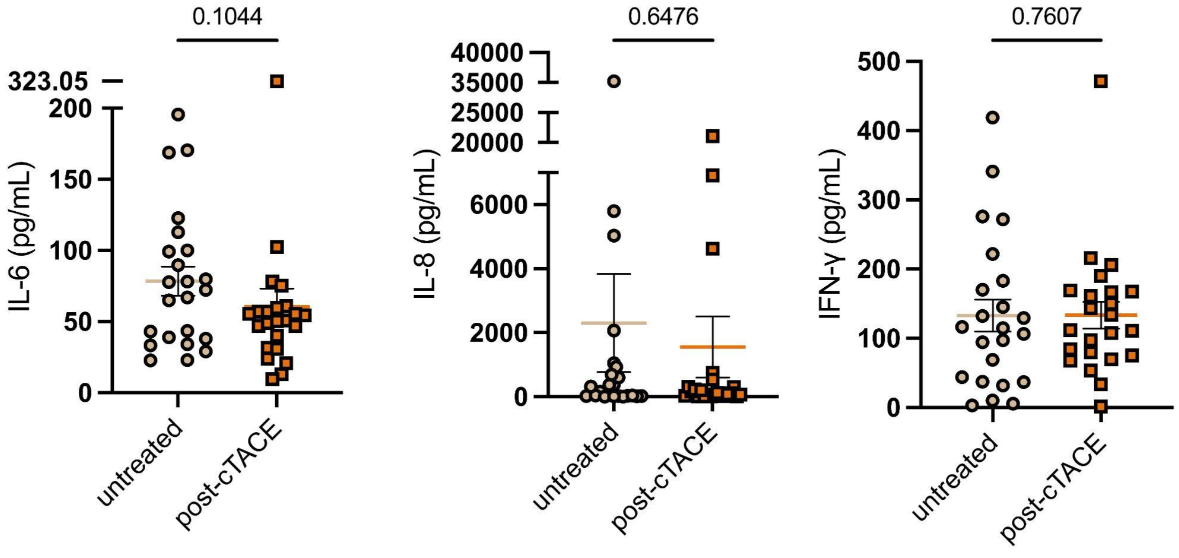

Supplementary Figure S2. Local immune landscape comparison between untreated (iBT) and post-cTACE.

Legend: Comparison of cytokine levels in tumor interstitial fluid (TIF) between untreated and cTACE-treated tumors. IL-6, IL-8, and IFN-γ levels showed no statistically significant differences between untreated and post-cTACE tumors ($p = 0.1044$, $p = 0.6476$, and $p = 0.7607$, respectively). Data were presented as mean ± SEM. Statistical analysis was performed using the Mann–Whitney U test.

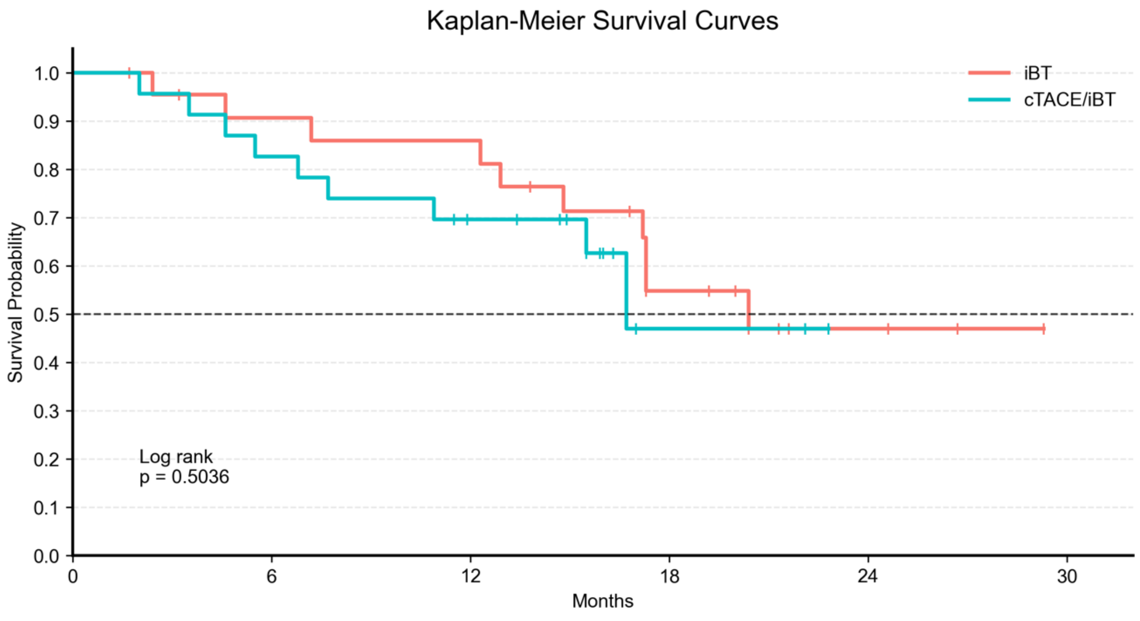

Number at risk (censored)

	0	6	12	18	24	30
iBT	23 (0)	19 (2)	18 (2)	9 (5)	3 (10)	0 (13)
cTACE/iBT	23 (0)	19 (0)	14 (2)	2 (12)	0 (14)	0 (14)

Supplementary Figure S3. Kaplan-Meier overall survival according to treatment group.

Legend: Kaplan-Meier survival curves comparing overall survival (OS) between patients treated with interstitial brachytherapy alone (iBT) and those treated with conventional transarterial chemoembolization followed by interstitial brachytherapy (cTACE/iBT). Time was displayed in months.

Survival distributions were compared using the log-rank (Mantel–Cox) test. Tick marks indicate censored observations. The number of patients at risk (with censored observations in parentheses) at predefined monthly time points is shown below the curves. No statistically significant difference in OS was observed between treatment groups (log-rank p = 0.5036).

References:

1. Lencioni R, Petruzzi P, Crocetti L. Chemoembolization of Hepatocellular Carcinoma. Semin Interv Radiol. 2013; 30: 003–11.
2. Ricke J, Wust P, Stohlmann A, Beck A, Cho CH, Pech M, et al. CT-guided interstitial brachytherapy of liver malignancies alone or in combination with thermal ablation: phase I–II results of a novel technique. Int J Radiat Oncol. 2004; 58: 1496–505.
3. Bretschneider T, Ricke J, Gebauer B, Streitparth F. Image-guided high-dose-rate brachytherapy of malignancies in various inner organs – technique, indications, and perspectives. J Contemp Brachytherapy. 2016; 3: 251–61.
4. Chernyak V, Santillan CS, Papadatos D, Sirlin CB. LI-RADS® algorithm: CT and MRI. Abdom Radiol. 2018; 43: 111–26.
